# HMMR has oncoprotein-like properties in neuroblastoma cells and high *HMMR* expression has independent prognostic potential in neuroblastomas

**DOI:** 10.1038/s41598-025-23141-5

**Published:** 2025-11-18

**Authors:** Christina Karapouliou, Elisyazaviera M. Faizul, Vinothini Rajeeve, Pedro R. Cutillas, Andrew W. Stoker

**Affiliations:** 1 Developmental Biology and Cancer Department, Great Ormond Street Institute of Child Health, University College London, 30 Guilford Street, London, WC1N 1EH UK; 2 Centre for Genomics and Computational Biology, Barts Cancer Institute, Queen Mary University of London, London, UK

**Keywords:** Neuroblastoma, HMMR, Phosphoproteomics, Hyaluronic acid, MTOR, DNA damage repair, Biochemistry, DNA damage and repair, Proteomics, Cell growth, Cell migration, Cell signalling, Cancer, Oncogenes, Paediatric cancer

## Abstract

Neuroblastoma (NB) is a devastating childhood cancer where most tumours have no clear oncogenic driver. We aimed to define whether HMMR, an oncogene-like protein in several cancers, harbors similar potential in neuroblastoma cells. HMMR is a hyaluronic acid (HA) receptor and a mitotic microtubule regulator. We show that high *HMMR* expression does not correlate well with *MYCN* driver expression and moreover statistically *HMMR* is an independent prognostic indicator of poor survival in NB patients. In cultured KELLY neuroblastoma cells, removal of the HMMR protein suppresses proliferation, motility and clonogenic capacity, while xenografts of HMMR-deficient cells imparted longer animal survival compared to wild type cells. Loss of motility in culture was compensated by addition of exogenous HA, suggesting that HMMR signaling is at least partly under HA control. Through an unbiased phosphoproteomic analysis, we also found that signaling downstream of MAPK1/2 was disrupted after loss of HMMR. In addition, RPS6 and p70S6 kinase were hypophosphorylated, while the DNA damage response (DDR) proteins such as CHK2 and TP53BP1 were significantly hyperphosphorylated. We thus provide, for the first time, evidence that HMMR does have oncoprotein-like properties in neuroblastoma cells and that *HMMR* expression has potential as a prognostic marker. Moreover, initial biochemical analyses highlight a potential influence for HMMR in MTOR and DDR pathway regulation.

## Introduction

Neuroblastoma is a developmental cancer of early childhood, arising from sympathoadrenal precursors of the neural crest^1^. Tumour cells are highly heterogenous and this heterogeneity contributes to the high relapse rate and poor survival of high-risk patients, representing a stubborn clinical challenge. Although the common oncogenic drivers found in adult human cancers are infrequent upon presentation in neuroblastoma^1,2^, several other drivers are known including *MYCN* gene amplification and activations of the tyrosine kinase ALK, or tyrosine phosphatase PTPN11^1^. Nevertheless, 75% of tumours have no clear oncogenic drivers^1,3^.

In our search for potentially new drivers with oncoprotein-like behaviour, we have examined the roles of HMMR, also known as RHAMM and CD168^4^, in neuroblastoma cells. HMMR may be of interest in a neural tumour because it has been implicated in neurite extension processes in neuronal cell lines, including in a hybrid neuroblastoma/glioma line NG108-15^5^, and HMMR loss-of-function generates neurodevelopmental defects in vertebrate embryos^6^. HMMR also has oncogenic roles in several other human cancer systems, sustaining cell proliferation, survival and migration in cells derived from cancers of brain, lung, ovary, prostate, head and neck and breast^7–10^. HMMR is a cell surface hyaluronic acid (HA) receptor^11^. HA and its catabolized products can promote cell proliferation and survival, motility and metastasis in tumour cells^12^, interacting with cells through an HMMR/CD44 complex and signaling through ERK, AKT, SRC, Rho GTPases and FAK^4,8,11–14^. Interestingly, HHMR also acts in the nucleus, binding to microtubules and centrosomes and regulating mitotic spindles and chromosomal stability through interactions with DYNLL1 complexes, CHICA and BRCA1^4,15^. HMMR also localises TPX2 to centrosomes, maintaining spindle pole assembly^16,17^. It operates through the release of TPX2 after HMMR degradation by BRCA1, leading to Aurora kinase A (AURKA) activation and BRCA1 phosphorylation^16^.

*HMMR* is strongly expressed in neuroblastomas and we hypothesised that it may have pro-oncogenic potential corresponding to that seen in other cancer models. Using in silico analysis we examined the relationship between high *HMMR* expression and prognostic outcomes in neuroblastoma tumours. At a cellular level we targeted *HMMR* for inactivation using CRISPR/Cas9 in the KELLY neuroblastoma cell line. Our analyses show that HMMR is indeed a promoter of several parameters of tumour cell behaviour and that these are variably affected by HA ligands. Lastly, we used phosphoproteomics to begin to explore the potential biochemical roles of HMMR in neuroblastoma cells, confirming that it modulates ERK signaling and potentially also MTOR and DNA damage response (DDR) pathways.

## Results

### HMMR as an independent prognostic marker in neuroblastoma

**Fig. 1:**
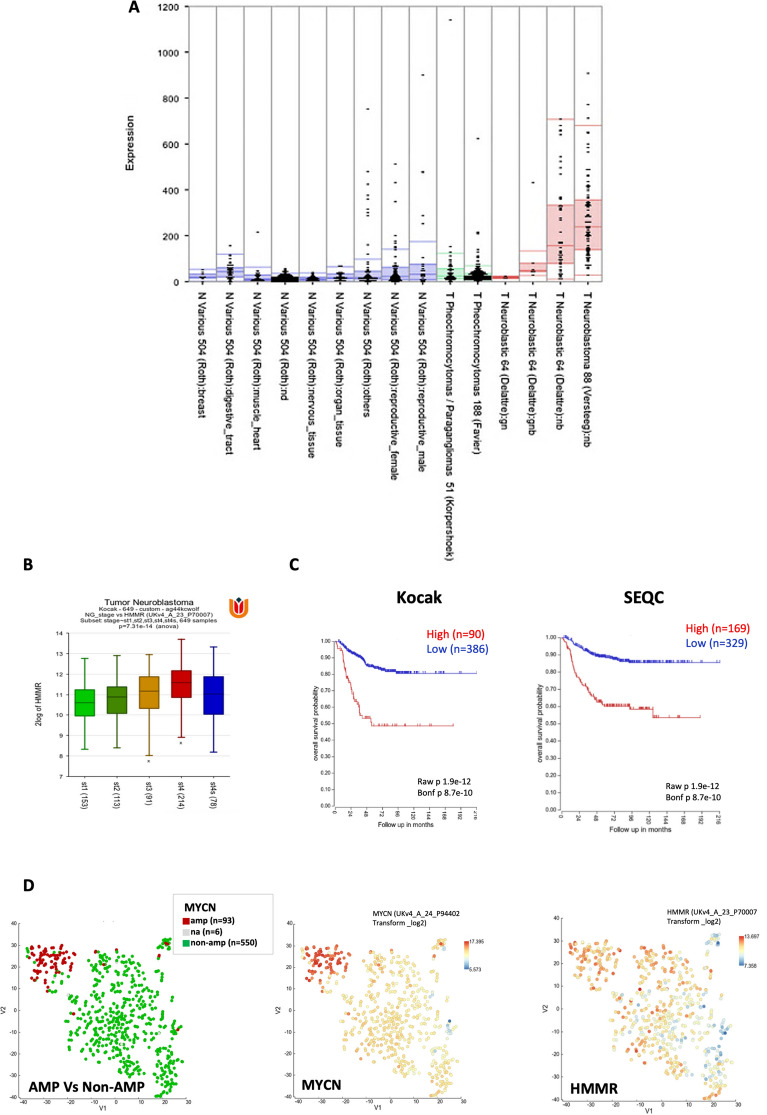
*HMMR* expression and neuroblastoma clinicopathological features. (**A**) The expression of HMMR in normal and neuroblastoma tissues examined by R2 genomics platform. Normal tissues (blue) are divided in 9 groups and pheocytochromocytomas/paragangliomas (green) in 2 groups. Neuroblastic tumours (red) are divided in 4 groups, from benign ganglioneuromas, ganglioblastomas to aggressive metastatic neuroblastomas. The authors and sample numbers are also shown. Grade staging (**B**) and overall survival (**C**) are depicted for the Kocak and SEQC datasets. (**D)** t-SNEA maps analysis on the Kocak dataset performed in R2, for *MYCN*- amplified or non-amplified group (left panel), *MYCN* expression (middle panel) and *HMMR* expression (right panel).

Although high *HMMR* expression has been associated with cancer progression^18^, this is yet to be investigated for neuroblastomas. *HMMR* expression was elevated in neuroblastomas compared to normal tissues, benign ganglioblastomas and neural crest-derived tumour pheochromocytoma (Fig. 1A and Supplementary Fig. S1). Moreover, *HMMR* is ranked in the top 1–5% overexpressed genes among those in the HA signaling axis, HA binding molecules and those associated with cell motility (Supplementary Fig. S1). This supports a possible role of the *HMMR* gene in the establishment or progression of neuroblastoma.

To further explore the role of HMMR in neuroblastomas, we examined the association between *HMMR* expression and tumour staging. Higher *HMMR* expression associated strongly with increased INSS tumour grade (Fig. 1B). We also found that elevated *HMMR* expression correlated significantly with poor overall survival (OS) in patient datasets analysed in the R2 platform (Fig. 1C). In t-SNEA maps, elevated *HMMR* expression partially overlaps with *MYCN*-amplified patient groups (AMP), but shows a somewhat differential expression pattern between patient data points, and it is also expanded to the non-AMP group (Fig. 1D). A Cox univariate and multivariable logistic regression analysis was performed on the SEQC dataset, demonstrating that *HMMR* expression, but not other HA-related pathway genes, is an independent risk factor for neuroblastoma patients (Table 1). To clarify the potential biological functions of HMMR we examined the genes that show positive correlations with *HMMR* expression in 4 tumour datasets (Supplementary Fig. S2). With this 2581 gene set, the *HMMR* co-expression signature again correlated with poor OS in neuroblastoma (Supplementary Fig. S2).

**Table 1:** Univariate and multivariable Cox regression of the prognostic covariates in patients with NB (n = 490, SEQC patient dataset from R2 Genomics). See Methods for full details.

Variable	HR (95%CI)	P value
Univariate		
HMMR	1.064 (1.049—1.078)	< 0.0001
CD44	0.9957 (0.9945 – 0.9969)	< 0.0001
HAS1	0.9921 (0.9232 – 1.048)	0.8036
HAS2	0.9766 (0.9217 – 1.018)	0.3474
HAS3	1.267 (1.203 – 1.332)	< 0.0001
HYAL1	0.7030 (0.5983 – 0.8210)	< 0.0001
HYAL2	1.033 (1.018 – 1.046)	< 0.0001
HYAL3	1.170 (1.085 – 1.245)	< 0.0001
MYCN	7.246 (4.900 – 10.71)	< 0.0001
INSS (1)	0.01261 (0.0007151 – 0.05674)	< 0.0001
INSS (2)	0.08056 (0.02458 – 0.1935)	< 0.0001
INSS (3)	0.3804 (0.2065 – 0.6497)	0.0009
INSS (4 s)	0.1193 (0.03639 – 0.2865)	< 0.0001
Age of diagnosis	1.000 (1.000 – 1.000)	< 0.0001
Multivariable		
HMMR	1.035 (1.010 – 1.058)	0.0041
CD44	0.9996 (0.9984 – 1.001)	0.4772
HAS3	1.049 (0.9677 – 1.131)	0.2289
HYAL1	0.9271 (0.8090 – 1.024)	0.2316
HYAL2	1.006 (0.9885—1.022)	0.5112

### HMMR promotes neuroblastoma cell proliferation

**Fig. 2:**
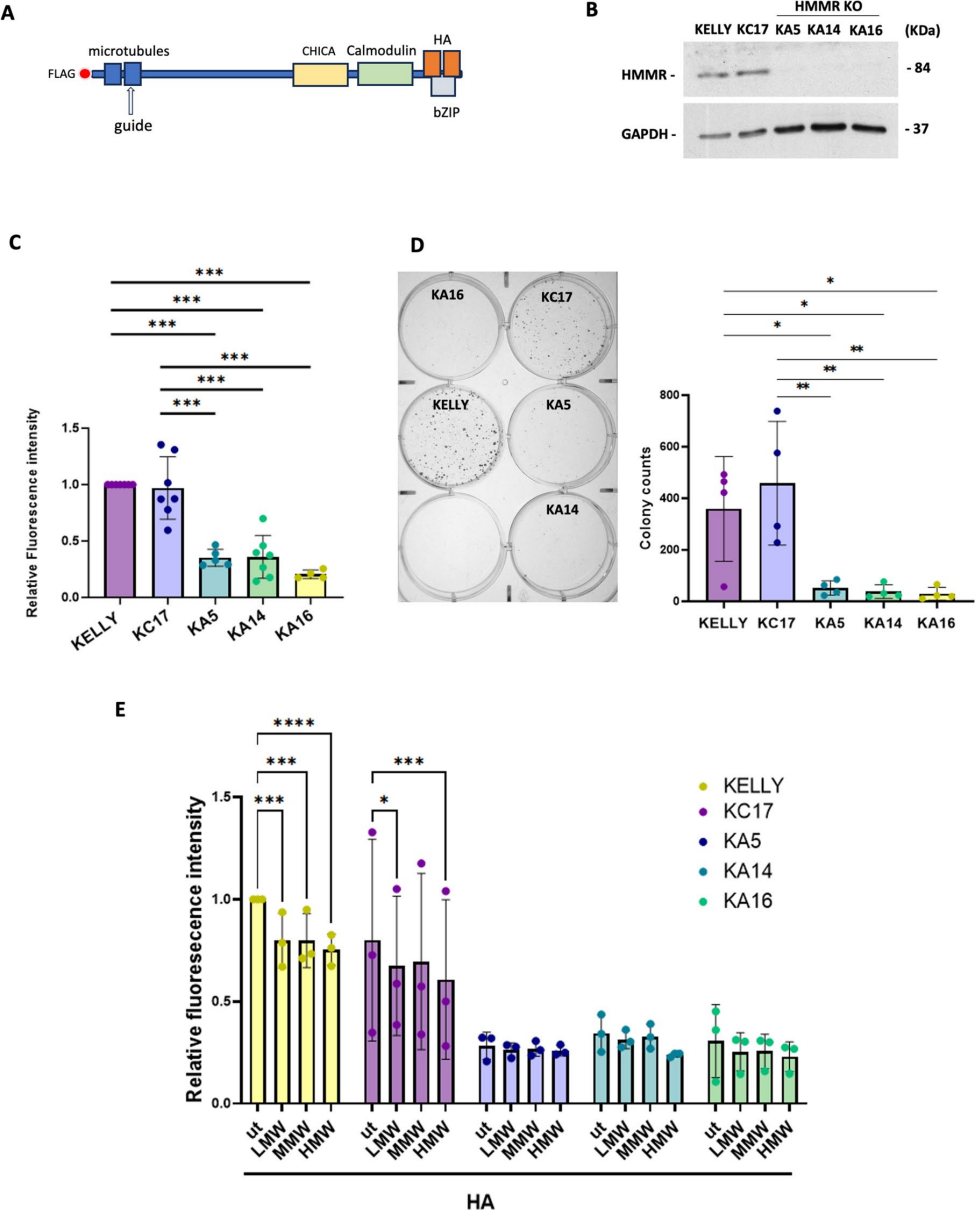
HMMR depletion suppresses proliferation and clonogenicity. (**A**) Schematic of the HMMR protein, with interaction domains shown for microtubules, HA, CHICA and Calmodulin, plus the carboxy-terminal bZIP region^52^. The guide RNA target site used in CRISPR/Cas9 is shown. (**B**) Immunoblot analysis of HMMR in parental KELLY cells and the clones generated by CRISPR/Cas9. (**C**) Cell proliferation assay using resazurin, normalized to growth of KELLY cells (n = 4–7). (**D**) clonogenic assay quantified from 6-well plate assays (example plate image shown, crystal violet staining) (n = 4). In C and D data are expressed as a mean ± SD; *p < 0.05, **p < 0.005, ***P ≤ 0.0005. (**E**) Parental KELLY, KC17 and HMMR KO clones were treated with HA in various sizes, LMW, MMW and HMW for 6 days and cell proliferation was assessed. Data were normalized to untreated (ut) KELLY cells and then expressed as a mean ± SD (n = 3). Two-way anova was performed; *p < 0.05, ***P ≤ 0.001, ****P ≤ 0.0001.

Equipped with these prognostics data, we next directly examined the cellular function of HMMR in neuroblastoma cells. Using CRISPR/Cas9 in KELLY cells (strong *HMMR* expressors) we created out-of-frame mutations in a region encoding the HMMR N-terminus (Fig. 2A). Three *HMMR* knock-out (KO) subclones were identified: KA5 (1 bp homozygous insertion), KA14 and KA16 (1 bp homozygous deletions; these are not proven to be independent subclones) (Supplementary Fig. S3). A further subclone, KC17, arose from the CRISPR/Cas9 selection process, but *HMMR* was not targeted with indels; we have not tested for other off-target alterations. The initial rationale for including this KC17 in the study was thus as a form of control for the CRISPR/Cas9 treatment process. It was confirmed that HMMR protein was absent from KO subclones KA5, KA14 and KA16, but was retained in KC17 and parental KELLY (Fig. 2B). The subcloned lines were all morphologically similar to parental KELLY (Supplementary Fig. S3). HMMR depletion in KA5, KA14 and KA16 inhibited their proliferative expansion compared to KELLY and KC17, agreeing with a similar role in other cancer cell types (Fig. 2C)^7,8^. Moreover, low density growth assays showed a strong reduction in colony forming ability in cells lacking HMMR (Fig. 2D), indicating a loss of clonogenic capacity.

HMMR is known to act as a surface co-receptor for HA, a prevalent glycosaminoglycan in extracellular matrices. HA can control cell proliferation in other cancer types, and different sizes of exogenous HA can have distinct effects^19^. To determine if KELLY proliferation is influenced by exogenous HA in an HMMR-dependent manner, cells were treated with either high, medium or low molecular weight (HMW, MMW or LMW) HA forms. We initially performed a dose-titration with a range of HA from 12.5 μM to 400 μM, to assess cell growth (data not shown). As the growth effect was almost the same among the different doses, we chose 400 µM for the proliferation and subsequent experiments with the aim to evaluate the maximum possible effect of HA on cell behavior.

Most forms of HA resulted in a mild, but statistically significant inhibition of the growth of KELLY and KC17 cells (Fig. 2E). However, these inhibitory effects were lost in *HMMR* KO cells, which were already growth-suppressed (Fig. 2E). These data suggest that control of KELLY proliferation could be at least in part dependent on HMMR as a co-receptor for HA signals.

### Loss of HMMR expression suppresses cell migration

**Fig. 3:**
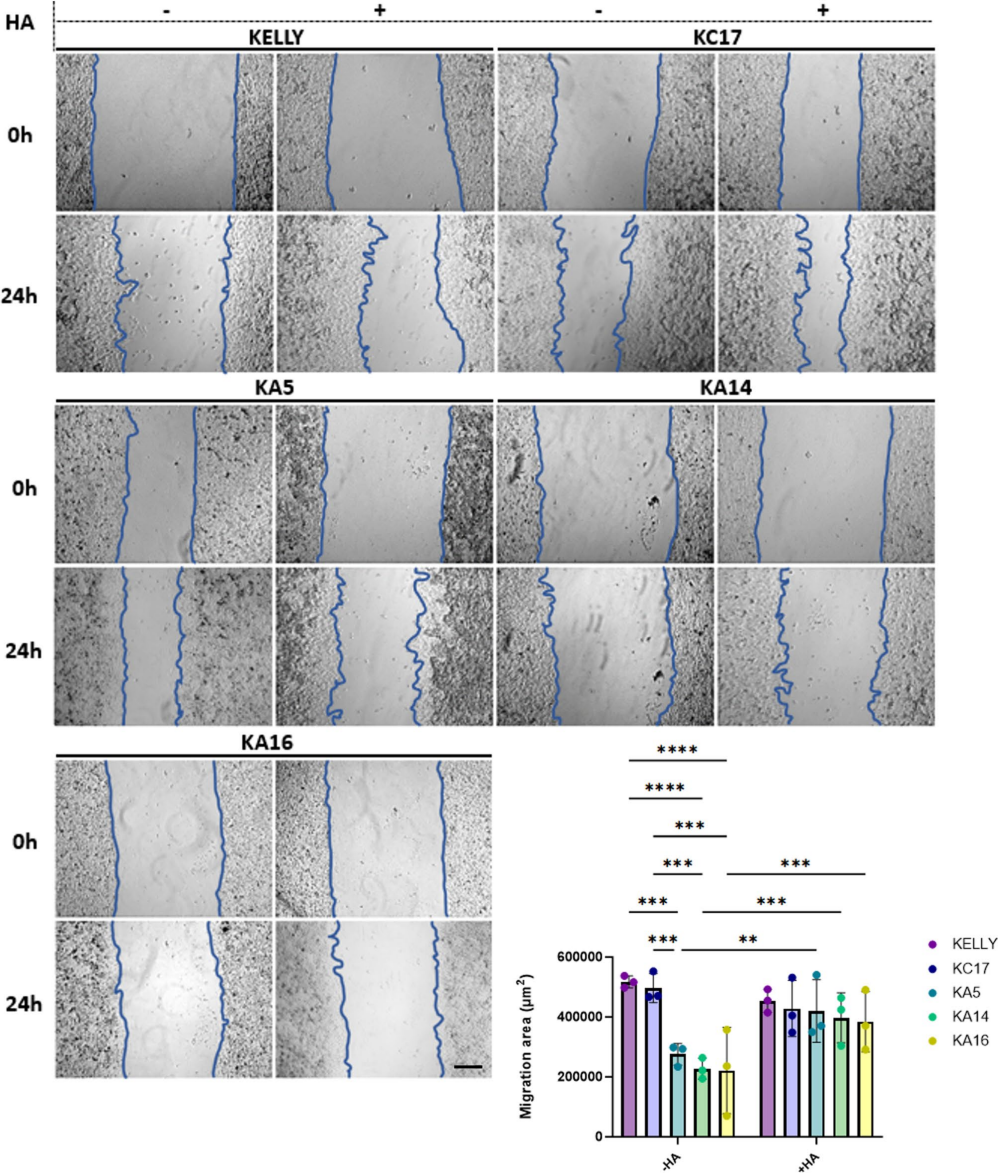
HMMR loss suppresses cell migration, but this is restored by exogenous HA. Parental KELLY, KC17 and *HMMR* KO clones were subjected to scratch assays for 24 h with or without addition of exogenous HMW HA (HA). The areas covered by migrating cells is expressed in µm^2^. Data are expressed as a mean ± SD (n = 3); **p < 0.005, ***P ≤ 0.0005, ****P ≤ 0.0001. Scale bar = 200µm.

HMMR can promote cell migration in a range of cell types^8,19,20^. We assessed if HMMR mediates migration in KELLY cells, by comparing the behaviour of wild type and mutated *HMMR* cells in wound healing assays. We also asked again if any effects seen could be further influenced by exogenous HA. In this initial study, HMW was used given that it had an equivalent, if not slightly greater inhibitory influence than the other forms in the growth assay. Using scratch assays to assess wound closure, it was evident that cells lacking HMMR migrated about half the speed of cells expressing HMMR (Fig. 3). When HMW HA was added to the media of HMMR-expressing cells, the rate of migration decreased slightly but not significantly. In contrast, HMMR-deficient cells recovered their migratory ability after the addition of soluble HMW HA, back to near control cell levels. These data indicate that although HMMR is required for maximal motility in KELLY cells, its loss can be compensated through a migration-stimulating effect of exogenous, high mass HA, possibly through other HA receptors.

### HMMR supports optimal tumour growth in vivo

**Fig. 4:**
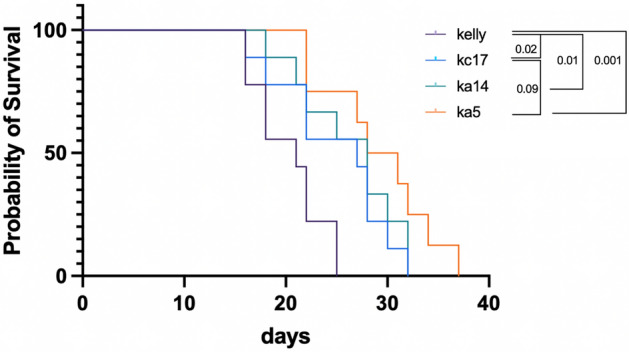
HMMR depletion reduces tumour growth. Survival assay of mice injected with parental, KC17 and *HMMR* KO clones. The length of time in days post-injection of animal termination is shown as a Kaplan–Meier plot. Pairwise comparison is made between tumour types using a Log-rank (Mantel-Cox) test and p-values are shown (KC17 vs KA14, p = 0.54).

To test the tumour-supporting potential of HMMR, cells were subcutaneously injected into NSG mice and tumour growth was monitored. The survival of mice harboring HMMR-deficient KA5 and KA14 tumors was significantly prolonged compared to parental KELLY (Fig. 4), corroborating the tumour-supporting role of HMMR. Despite the similar behavior of KC17 to the parental KELLY cells in other assays, this cell line showed tumour outgrowth similar to KA14. KC17 therefore does not behave identically to KELLY parental cells and is not therefore an optimal control for this assay. Below, our phosphoproteomic analysis also identified significant differences between KC17, KELLY and the mutant lines.

### Phosphoproteomic analysis of the effects of HMMR loss

**Fig. 5:**
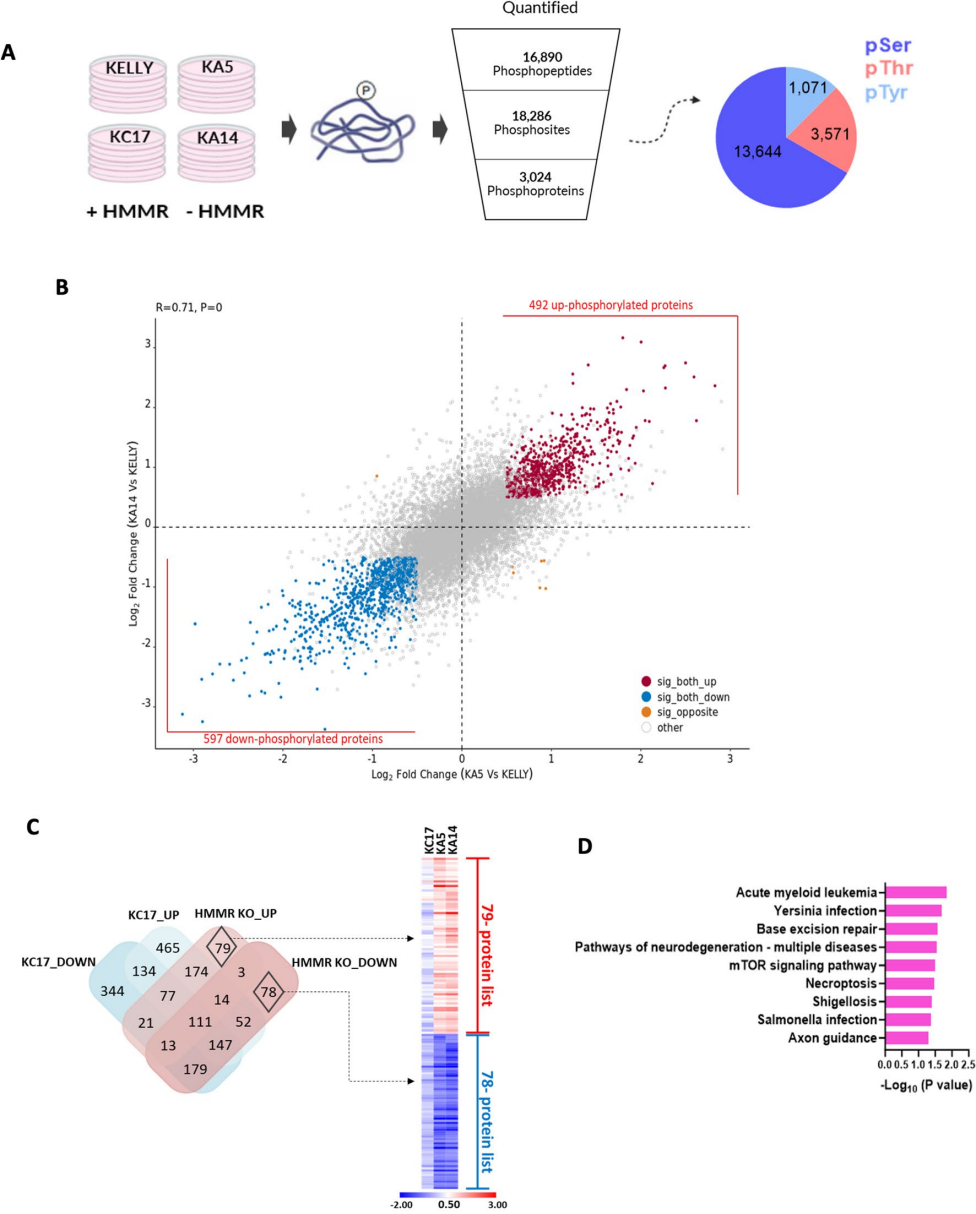
Phosphoproteomic profiling. (**A**) Schematic representation of the phosphoproteomic pipeline. KELLY cells expressing HMMR (parental and KC17) or not (KA5 and KA14) were harvested and phosphoproteomic analysis performed. Pie chart shows the number of identified phosphosites; N = 4 biological replicates. (**B**) Correlation plot of phosphopeptide differences seen between KA5 vs KELLY and between KA14 vs KELLY. Each spot is a phosphorylation site. Thresholds were differential phosphorylation from KELLY of Log_2_ -0.5/ + 0.5 and p-value of 0.05 or below. Proteins with statistically significant up- and down- phosphorylation are marked with red and blue, respectively, whereas orange peptides have a statistically significant, opposite correlation. (**C**) Phosphoproteomic HMMR signature. A Venn diagram showing overlaps between significantly altered phosphopeptides found in both KA5 and KA14 (collectively logFC ≥ 0.5, logFC ≤ 0.5, p ≤ 0.05, from Fig. 5B), and altered sites in KC17 cells (p ≤ 0.05). The unique 79- and 78- proteins in response to HMMR depletion are marked with black diamonds, generating the HMMR phosphoproteomic signature. A heat map of the signature in comparison to KC17 is presented. Colours represent fold change over parental proteins expressed as Log2. (**D**) KEGG enrichment analysis of the HMMR signature; P > 1.3 of -Log_10_.

In starting to define potential HMMR signaling pathways, we performed mass spectrometry (MS)-based quantitative phosphoproteomics on KELLY, KC17, KA5 and KA14 (Fig. 5A). In comparison to KELLY cells, phosphorylation significantly increased in 878 (KA5) and 1310 (KA14) peptides (Supplementary Fig. S4; P < 0.05, FDR), and decreased in 1013 and 1937, respectively. KA5 and KA14 had closely correlating phosphorylation profiles (R = 0.71, P < 0.0001; Fig. 5B) and overlapping principal component analysis (PCA) patterns (Supplementary Fig. S4), suggesting their signaling was affected relatively similarly.

**Fig. 6:**
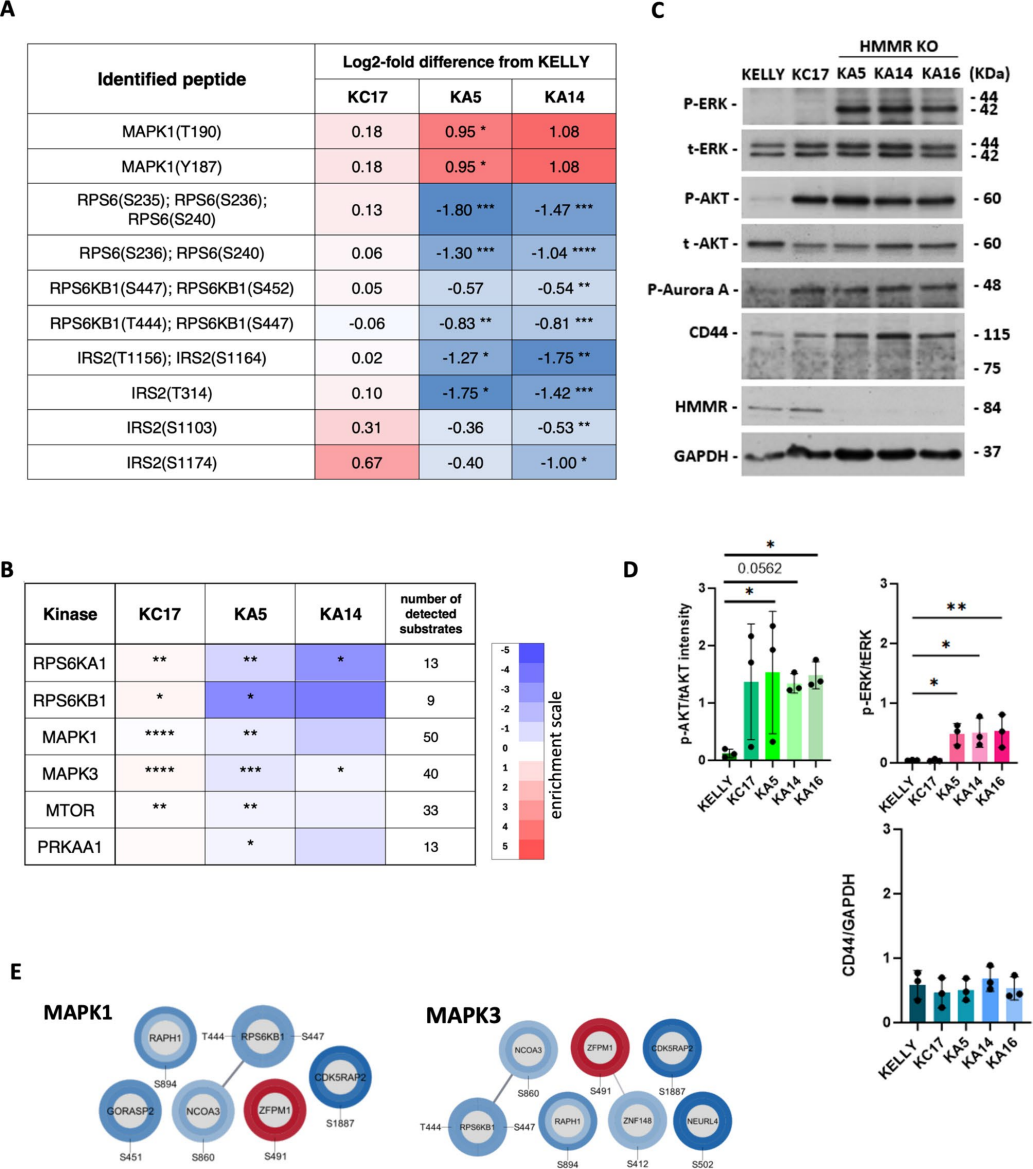
Functional activity of the HMMR phosphoproteomic signature. (**A**) Phosphopeptide differences between cell lines and KELLY, observed in components of the MTOR regulatory network. Red and blue show log_2_-fold increased or decreased phosphorylation, respectively; white is no change (* P < 0.05, ** P < 0.01, *** P < 0.001). (**B**) KSEA analysis of differentially phosphorylated peptides in the whole dataset, showing those most relevant to MTOR signaling. (**C**) Immunoblot analysis of cell lines for phosphorylated ERK (P-T185/P-Y187 (ERK2) or P-T202/P-Y204 (ERK1)), AKT (S473), and Aurora A as well as HMMR and CD44 (n = 3); original data in Supplementary Fig. S8. (**D**) Protein signals were normalised to either their respective total proteins or GAPDH, and then expressed as a mean ± SD; *P ≤ 0.05, **p < 0.005. (**E**) Examples of MAPK3 (ERK1) and MAPK1 (ERK2) substrates are shown as protein networks based on STRING and visualised in Cytoscape. Inner and outer rings represent kinase activity status (z score). Red and blue colour indicate increased or decreased kinase activity, respectively, with greater colour intensity reflecting greater z scores.

Although KC17 was intended here as an *HMMR*-expressing control, these cells clustered separately from parental and HMMR-depleted cells in a PCA analysis (Supplementary Fig. S4) indicating that the cells are not identical biochemically to these other cells, and data from KC17 should be treated with caution. To define an HMMR-specific signature, we generated a conservative list of 157 peptides (79 upregulated plus 78 downregulated) specifically altered in HMMR-deficient cells compared to KELLY, and excluding KC17 changes, filtering additionally for Log_2_FC > 0.5 or < -0.5 (Fig. 5C , p≤ 0.05, FDR). Initial KEGG analysis showed significant enrichment for pathways including that of MTOR (Fig. 5D). There was also an indication that infection pathways are affected, which is of interest given that HMMR has been implicated in the innate immune response^21–23^. For the MTOR pathway, log_2_-fold phosphorylation changes of key peptides in the MTOR network showed a general decrease, indicating partial suppression of MTOR signaling (Fig. 6A and Supplementary Fig. S5). In particular, RPS6 showed reduced phosphorylation of RPS6KB1 (p70S6K) target sites, while RPS6KB1 itself showed reduced phosphorylation on amino acids targeted by ERK. Curiously, phosphorylation of MAPK kinase ERK2 (MAPK1) itself was instead increased on Thr190 and Tyr187 in HMMR-depleted cells (Fig. 6A, C, D; the antibody recognizes equivalent of P-Thr185 and P-Tyr187 in ERK2). KSEA on the signature set revealed diverse kinases among both the up- and down-regulated pools (Supplementary Fig. S5). The ERK1 (MAPK3) pathway reached statistical significance for clones KA5 and KA14, and ERK2 (MAPK1) was close to significance (p 0.057); both, however showed down-regulated ERK downstream pathways. We also applied KSEA to the differentially phosphorylated peptides in the entire dataset, again observing modest downregulation of ERK pathways (Fig. 6B). In assessing the activation status of the direct substrates of ERK, all except ZFPM1 were downregulated for both ERK1 and ERK2, confirming partial downregulation of ERK signaling (Fig. 6E). IPA analyses also confirmed this (Supplementary Fig. S5, blue lines, and Supplementary Fig. S6). We thus conclude that HMMR depletion counterintuitively increases ERK2 phosphorylation, but suppresses the activity of the downstream overall ERK cascade.

Given that MTOR signals may be influenced by HMMR, we looked for upstream regulation of AKT. In immunoblots AKT showed an inconsistently increased phosphorylation of activation site S473 in HMMR depleted cells, but this also occurred in KC17 (Fig. 6C, D). The S473 peptide was not however identifiable in the phosphoproteomic dataset. Our examination of other common substrates in the proteomics data revealed no clear pattern of AKT activation or inactivation. Thus it is most likely that S6KB1 inactivation after loss of HMMR is not due to AKT suppression.

We found that IRS2, but not IRS1, is also hypophosphorylated after loss of HMMR (Fig. 6A). This is potentially relevant given it can be upstream of MTOR and downstream of the neuroblastoma oncoprotein ALK^24^.

Other components related to HMMR activities, including phosphorylated Aurora Kinase A and CD44, do not appear to alter in level after HMMR removal (Fig. 6C, D). The predominant CD44 isoform seen in KELLY is CD44v3, which, along with the standard 85kDa isoform, has previously been shown to associate with HMMR in breast cancer cells^8^ and head and neck cancer^25^.

### DNA damage response proteins are hypophosphorylated after HMMR loss

**Fig. 7:**
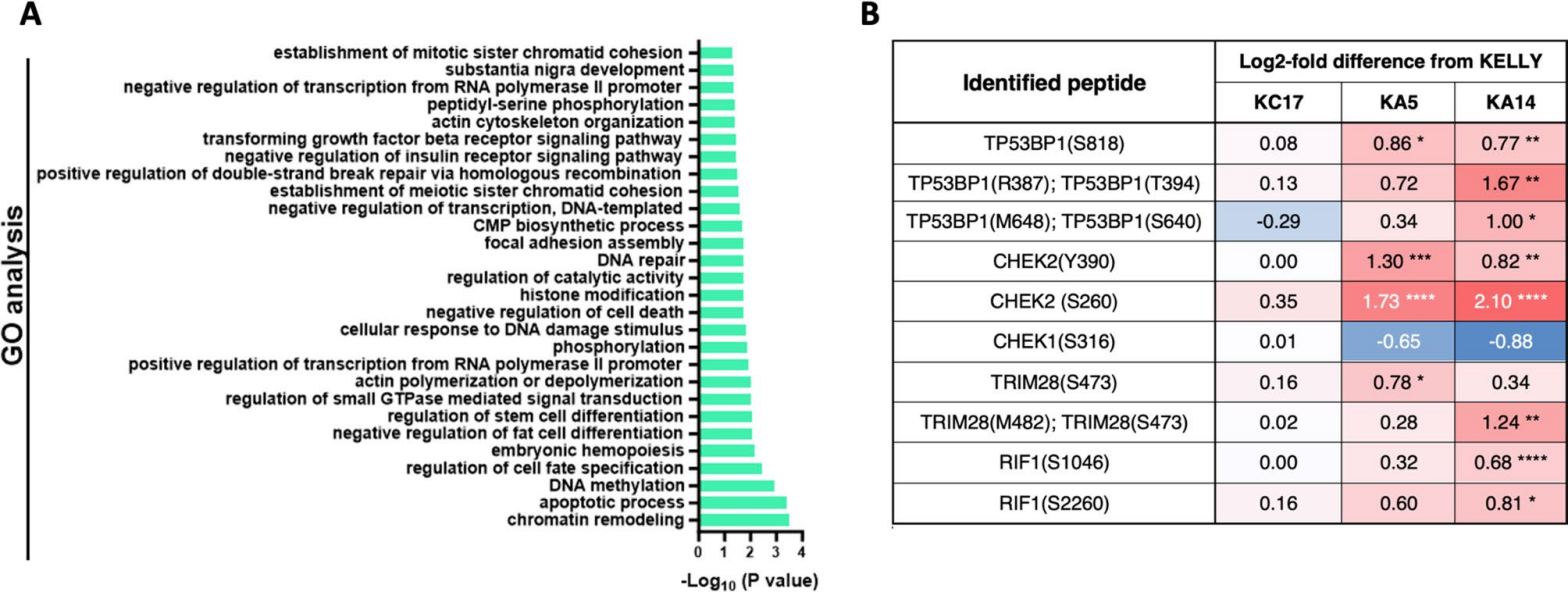
HMMR and the DNA damage response. (**A**) ShinyGO enrichment analysis of the HMMR phosphoproteomic signature is shown, with pathways all P > 1.3 of -Log_10_. (**B**) Phosphopeptide differences between cell lines and KELLY, observed in components of DDR pathways (* P < 0.05, ** P < 0.01, *** P < 0.001, **** P < 0.0001). Red and blue show log_2_-fold increased or decreased phosphorylation, respectively; white is no change.

GO term analysis on the HMMR signature peptides revealed processes including double-strand break repair of DNA, DNA repair, and responses to DNA damage stimuli (Fig. 7A). Furthermore, the HMMR-co-expression dataset also picked up several aspects of DDR in a GO analysis (Supplementary Fig. S2). Examination of the phosphoproteomic data for DDR proteins revealed several with significantly increased phosphorylation after HMMR depletion (Fig. 7B). These include 53BP1 and RIF1, which together bind double-strand DNA breaks and influence non-homologous end joining (NHEJ)^26,27^. These are not known ATM or ATR target sites and their role in modulating DDR is currently unclear. KAP1 (TRIM28) also shows a potential hyperphosphorylation on S473, a stimulatory site targeted by CHK1 and CHK2 after DNA damage^28,29^; this alteration is statistically significant only in KA5. CHK2 itself shows increased phosphorylation on Y390 in KA5 and KA14, but not KC17; Y390 phosphorylation is necessary for CHK2 kinase activity^30^. S260 in CHK2, another autophosphorylation site^31^, also shows hyperphosphorylation in KA5 and KA14. In contrast, some hypophosphorylation is seen on CHK1 S316, a potential autophosphorylation site next to ATR target S317^32^. Direct evidence for activation of ATM, ATR and BRCA1 is not evident in the dataset. Collectively, the phosphoproteomic data point to there being a restricted but significant perturbation in the DDR network after loss of HMMR.

## Discussion

HMMR has oncogenic potential in several cancer models, with our study being the first in neuroblastoma. High *HMMR* expression correlates strongly with poor prognosis and could be an independent risk factor for neuroblastoma patients. We also show in cultured KELLY cells that loss of HMMR leads to reduced proliferation, 2D colony formation and 2D migration. Xenograft analysis also suggests that HMMR is required for maximal tumour growth rate. Our initial, unbiased phosphoproteomic study of these cells indicates that HMMR is directly or indirectly influencing the phosphorylation of many proteins including ERK, IRS2, S6KB1 and S6K. Moreover, we have identified a further potential influence of HMRR in the cell’s DDR network. Overall, these data indicate that HMMR could be an unexplored driver of cancer cell behaviour in neuroblastoma cells with a broad signaling influence.

From neuroblastoma patient datasets, high *HMMR* expression can mark tumours as higher risk and *HMMR* expression represents a risk factor independent of *MYCN*. Although HMMR is an HA receptor, other HA-signaling genes, including *CD44*, do not show prognostic significance, suggesting that HA signaling per se may not be a sufficient influence behind HMMR’s pro-oncogenic actions in these tumours.

HMMR-deficient KELLY cells have reduced proliferation rates, agreeing with what is observed in other tumour cell types^9,10,33^. HMMR acts alongside CD44 as a cell surface HA receptor to generate ERK signaling, although depending on the size of HA used this signaling can be either promoted or inhibited (reviewed in^34^). In our study, exogenous HA of varying sizes modestly suppressed proliferation in KELLY cells, whereas cells lacking HMMR (already very growth suppressed) were not further growth-suppressed. The likely explanation of these findings is that endogenous HA normally drives ERK signals and proliferation in part through HMMR/CD44 complexes, and that exogenous HA can partially interfere with this. Once HMMR is removed from the system, however, both endogenous and exogenous HA may be hampered in their ability to modulate ERK through CD44 signaling, even though CD44 levels were normal in HMMR-deficient cells.

At clonogenic density the HMMR-deficient cells struggled to self-renew as 2D colonies. Our later study of HMMR-deficient IMR32 cells indicated a similar deficiency (Supplementary Fig. S7). This aligns with the proposed stemness-promoting capacity of HMMR in glioblastoma cells^9^. To counter this, however, *HMMR* expression in neuroblastoma tumours (from R2 analysis) does not correlate well with proposed neuroblastoma stem cell genes such as *NOTCH*, *GPRC5C* or *TRKB*^31^. HMMR’s clonogenic function may relate to its maintenance of ERK signaling, but this needs further investigation.

HMMR is also required for optimal motility in a wound repair assay of KELLY cells, corroborating similar findings in other cell types. HMMR cooperates with CD44 to promote cell motility through ERK, FAK and SRC^8,19,20^, and cells lacking HMMR can cause a deficit in this CD44-mediated signaling^35^. Our phosphoproteomic data reveal a similar correlation at least between motility and ERK signaling in neuroblastoma cells. Exogenous, HMW HA slightly decreased motility in KELLY cells, but surprisingly it rescued the migration deficit in HMMR-deficient cells. HA influences motility in other cells both positively and negatively, complicated by the HA size range^19,34,36^. One hypothesis here is that loss of HMMR blocks CD44’s ability to efficiently bind endogenous HA and the migration signals then falter. High levels of exogenous HMW HA, however, may be able to drive CD44 multimer formation, re-initiating the signaling required for motility^37^. This contrasts with exogenous HA’s inability to recover cell proliferation in HMMR-deficient cells. Given that different sized forms of HA have varied influences over cell motility^19,20,33^, our current experiment using only HMW HA in KELLY cells does have its limitations. Future motility studies could therefore benefit from the testing of smaller forms of HA.

Using xenografted tumours of our KELLY derivatives, our study supports the possibility that HMMR positively promotes KELLY tumour growth. This would currently concur with other cancer models where HMMR was able to support tumour growth^9,10^. One caveat was that the *HMMR*-intact line KC17 also showed slower growth than KELLY, however the proteomic data later showed that KC17 was biochemically non-identical to KELLY and the KO lines and so its behaviour in vivo is difficult to interpret (discussed further below). Further studies with other HMMR-depleted neuroblastoma cell lines would therefore be beneficial to reinforce our understanding of HMMR loss in the in vivo context. Moreover, the cellular studies could also benefit from HMMR rescue data. However, our two attempts to date to transfect and select for *HMMR*-encoding plasmids in the KO lines have not yielded *HMMR*-re-expressing cells, and so this awaits future investigation.

Given the seemingly context-dependent properties of the KC17 subclone, we need to expand on our reasoning for including it in this study. Our rationale was to use a cell clone that had come through the complete CRISPR/Cas9 transfection and selection process, but without HMMR targeting. Such a cell leaves HMMR intact, but we acknowledge that it may well have other off-target alterations that could subtly change its behaviour independently of HMMR. Nevertheless, we identified this one line from the process and used it to define some of the cellular and molecular differences between cells retaining and losing HMMR protein. While the culture assays showed KC17 to behave the same as parental cells, there appear to be context-dependent differences from KELLY revealed in vivo. The phosphoproteomics-based PCA analysis (Supplementary Fig. S4) also later showed that KC17 was non-identical to KELLY and to KA5/KA14. KELLY cells may be biochemically somewhat heterogenous or unstable, and this may lead to the subtle, context-related differences in behaviour between KC17 and KELLY, even though they retain HMMR. The potential basis of this is currently unclear. Thus, although KC17 exhibited some differences from parental cells, the data from this subclone were included particularly to highlight some of the clear differences between the *HMMR*-expressing and -non-expressing KELLY cells.

Phosphoproteomics has allowed us to start uncovering some of the potential signaling that HMMR can directly or indirectly modulate in neuroblastoma cells. Unexpectedly, MAPK1 (ERK2) was hyperphosphorylated in the KO cells, while ERK1/2 downstream signaling was significantly suppressed. The latter was predicted given the documented, stimulatory role of HMMR over ERK1/2^8,32^, but we cannot currently explain why ERK2 phosphorylation itself increases. HMMR is documented to bind directly to ERK1, but indirectly to ERK2^8^. There may be some compensatory effect operating through CD44, comparable to the increased HMMR signaling seen after CD44 loss in leukocytes^22^, based on increased HA levels. In addition, the documented complexes of HMMR and CD44 with ERK and MEK proteins^8^ may still activate ERK2 when HMMR is absent from the complexes in the specific context of KELLY cells, but this still appears insufficient to activate the ERK1/2 downstream pathway.

In KSEA and GO analyses, components of the MTOR network were also suppressed. MTOR is a central regulator of cellular responses to growth factors and cell stress, and integrates signals from ERK, AKT and other signals^38^. A reduction in MTOR signals, particularly those through RPS6KB1 and RPS6 that we observe, might explain in part the reduced proliferation and survival profiles of the HMMR-depleted cells. CD44 and MTOR also regulate each other’s actions in AML and breast cancer models^39^, indicating that HA signaling can also feed into this pathway. The observed reduction in IRS2 phosphorylation may also relate to the reduced MTOR signaling, although this is speculative given that the phosphosites we observe are not characterised. IRS2 nevertheless is of interest given that ALK uses IRS2 as an effector in neuroblastoma cells, possibly through AKT3^24^ and its signaling control and effects are distinct from IRS1^40^.

Alongside it’s HA receptor role, HMMR has well documented nuclear roles in spindle dynamics and interactions with BRCA1^4^. Given BRCA1’s central role in homologous DNA repair, it was interesting that loss of HMMR in KELLY cells led to hyperphosphorylation of some DDR regulators. These included 53BP1, KAP1, RIF1 and CHK2. Known regulatory phosphosites were altered on CHK2^30,31^ and KAP1^28,29,32^, while numerous phosphopeptides in 53BP1 remain to be functionally understood. This raises the hypothesis that HMMR is a direct or indirect modulator of DDR in neuroblastoma cells, a potentially new role for this protein. Loss of HMMR may disrupt spindle and chromosome dynamics in neuroblastoma cells, as seen in other cancer cells^15^, generating mitotic stress and DNA damage. However, we currently see no direct evidence of ATM or ATR activation or altered phosphorylation targets such as T68 in CHK2 or S824 in KAP1. Our observation of RPS6KB1 and RPS6 hypophosphorylation could also relate to DNA damage since DNA damage suppresses S6K1-mediated RPS6 phosphorylation in various tumour cells^41^. A new role for HMMR in DDR within neuroblastoma cells is therefore proposed, requiring further investigation.

To conclude, we show for the first time that high expression of *HMMR* and its product HMMR are statistically implicated in poor neuroblastoma patient outcomes and also in supporting several cancer cell behaviors in KELLY cells. Potentially new areas of HMMR influence include modulation of protein phosphorylation in the MTOR and DDR pathways, warranting further exploration. HMMR could thus form an unrecognised signaling hub in these tumour cells, opening avenues for future prognostic or therapeutic investigation.

## Materials and methods

### Cell culture

KELLY cells (CVCL_2092) were provided by Prof. Frank Speleman, University of Ghent. Cells were STR genotyped in 2015 by LGC Standards. Cells were cultured at 37°C in RPMI medium + GlutaMAX (ThermoFisher Scientific, Loughborough, UK) supplemented with 10% FBS (Life Technologies) and 100 U^.^mL^-1^ Penicillin, 0.1 mg mL^-1^ Streptomycin (Sigma-Aldrich, UK). The HA types used were low (Sigma-Aldrich 40,583; LMW, 5000–1000 Da), medium (Sigma-Aldrich 75,044; MMW, 150,000–300,000 Da) and high molecular weights (Sigma-Aldrich 51,967; HMW, 1.5–1.8 × 10^6^ Da). HA was dissolved in media at the final concentration of 400 μg/mL.

### Generation of *HMMR* knockout (KO) subclones

The *HMMR* gRNA in exon 5 (CGTGTTCTTCTACAGGAACG) was designed using Benchling (San Francisco, CA, USA; RRID:SCR_013955). Plasmid co-expressing Cas9 and gRNA was purchased from VectorBuilder (Neu-Isenberg, Germany) and transfected using Lipofectamine 2000 (Thermo Fisher Scientific, USA). Cells were incubated for 4–6 days with 1mg/ml puromycin, then single cell sorted using a MoFlo XDP sorter. DNA target regions were subjected to PCR amplification using 5’-GCAACAGAGCACAGAGCAAG-3’ and 5’-ACACCAGGCGATTCAGATTC-3’ and sequenced (Source Bioscience, UK). Sequence trace analysis was performed using the ICE ANALYSIS online tool (Synthego, v2.0; Synthego, CA, USA; RRID:SCR_024508).

### Cell assays

Two thousand cells were seeded in 96-well plates and cell viability was measured after 6 days with resazurin (Merck Life Science UK Ltd, Gillingham, UK). For the clonogenic assay, 400 viable KELLY cells were seeded in 6-well plates and incubated for 3 weeks. Cells were fixed using crystal violet (Sigma-Aldrich) in 25% Methanol. Colonies were counted manually using ImageJ software. For migration assays, cells were grown to confluency in 24-well plates and serum starved overnight. A 200µl pipette tip was used to make a scratch in the monolayer of duplicate wells and cells were then incubated in serum-free media with or without added 400μg/ml of HA for 24 h. Initial and final cell-free scratch areas were measured using the wound healing size plugin in ImageJ software^42^. Differences between the 0 and 24 h were expressed as migration area.

### Mouse xenografts

Animal usage fully complied with the guidelines and regulations under UK Home Office project licence (PP5675666), complying with the UK Home Office Guidance on the Operation of the Animals (Scientific Procedures) Act (ASPA) 1986. All experimental protocols were further approved by the UCL Biological Services Manager and complied with local animal husbandry regulations and procedures. The number of animals used and all methods are reported in accordance with ARRIVE guidelines. Female NSG mice (purchased direct from Charles River Laboratories, Sulzfeld, Germany) were injected subcutaneously in the flank with 2 × 10^6^ KELLY, KC17, KA5 or KA14 cells, suspended in a 1:1 PBS and Cultrex matrix (R&D Systems Inc., USA). Each injection group consisted of 3 animals per cell line (12 animals total) and injection groups were repeated three times. Mice were randomly assigned to groups and cell injections plus tumour measurements were performed in a blinded fashion. Euthanasia was administered using schedule 1 cervical dislocation as approved under ASPA, 1986. Tumor volumes (in mm^3^) were determined using digital calipers [length x (width)^2^/2].

### Immunoblotting

Cells were processed for immunoblotting as previously described^43^. Primary antibodies (Cell Signaling) were against: pan-CD44 (#3578; RRID:AB_2076463), pERK (#9106; RRID:AB_331768); tERK (#9102; RRID:AB_330744); pAKT (#4060; RRID:AB_2315049); tAKT (#9272; RRID:AB_329827), p-Aurora A (#3079; RRID:AB_2061481), GAPDH (#2118; RRID:AB_561053). Anti-HMMR GTX121502 (GeneTex; RRID:AB_11163915) was also used. Protein expression was quantified by densitometry on X-ray films using ImageJ software (RRID:SCR_003070).

### Phosphoproteomic study and analysis

Cells were lysed in 8 M urea with phosphatase inhibitors (10 mM Na_3_VO_4_, 100 mM b-glycerol phosphate and 25 mM Na_2_H_2_P_2_O_7_ (Merck Life Science UK Ltd)) as described previously^43^. Four biological replicates were subjected to mass spectrometry as described previously^43^. The data analysis was performed using the Limma R package (version 3.50.1; RRID:SCR_010943) and p-values were corrected using the qvalue package (version 2.26.0; RRID:SCR_001073) from Bioconductor as described previously^44^. To correlate the differentially expressed phosphoproteins of the HMMR-deficient cells, Pearson’s correlation performed using the online platform ‘ggVolcanoR’^45^. For the HMMR phosphoproteomic signature, proteins with fold changes of 0.5 and p ≤ 0.05 were considered statistically significant and differentially phosphorylated from controls (78 and 79 protein lists). The heatmap was generated using Morpheus (Broad Institute). For the Kinase substrate enrichment analysis (KSEA), peptide data were processed as previously described^46^ (Fig. 6B) and also with the KSEAapp tool^47^ (Supplementary Fig. S5). Networks were visualised in Cytoscape^48^ (RRID:SCR_003032) using STRINGAPP^49^ (RRID:SCR_025009) and OMICS VISUALISER^48^ plug-ins. Ingenuity Pathways Analysis (IPA) software (Qiagen, USA; RRID:SCR_008653) was used for upstream regulator analysis. The processed phosphoproteomics data are deposited with Mendeley Data and available at https://data.mendeley.com/datasets/6wr2tj8wr9/1.

### Tumour data and pathway analysis

Patient tumour data and Kaplan–Meier survival curves were obtained using the R2 genomics platform (http://r2.amc.nl; RRID:SCR_025770). Datasets used were: Roth (n = 504, GEO: GSE7307), Korpershoek (n = 51, GEO: GSE67066), Favier (n = 188), Delattre (n = 64, GEO: GSE12460), Versteeg (n = 88, GEO: GSE16476), Kocak (n = 649, GEO: GSE45547), SEQC (n = 498, GEO: GSE49710), NRC (n = 283, GEO: GSE85047) and TARGET-Asgharzadeh (n = 249, GEO: GSE85047). Comparison analyses between differentially expressed genes were also obtained from the Oncomine™ Platform (Thermo Fisher; RRID:SCR_007834) using the studies Albino Brain (n = 28, GEO:GSE7529) and Janoueix-Lerosey Brain (n = 64, GEO: GSE12460). Kyoto Encyclopedia of Genes and Genomes (KEGG) pathway and Gene Ontology (GO) enrichment analyses were conducted using DAVID^50^ (RRID:SCR_001881) and ShinyGO 0.80^51^ (RRID:SCR_019213).

### Statistical analysis

Statistical analysis and graphing was performed using GraphPad Prism 10.0 (RRID:SCR_002798). One-way and two-way (Dunnett post hoc analysis) ANOVAs were used. Cell proliferation data were statistically analysed as part of an experimental dataset with multiple treatments but here we show only the analysis relevant to this paper. A Cox regression model was used to test for the independent predictive ability of HMMR expression after adjustment for other significant factors: MYCN amplification, age, and INSS stages.

## Supplementary Information

Supplementary Information 1.

Supplementary Information 2.

## Data Availability

The processed phosphoproteomics data are deposited with Mendeley Data and available at https://data.mendeley.com/datasets/6wr2tj8wr9/1.

## Abbreviations

NB: Neuroblastoma
DDR: DNA damage repair
GO: Gene ontology
HA: Hyaluronic acid
KO: Knockout
KEGG: Kyoto Encyclopedia of Genes and Genomes
NHEJ: Non-homologous end joining
KSEA: Kinase substrate enrichment analysis
OS: Overall survival
AMP: *MYCN*-amplified
