# Supplementary material for: HMMR has oncoprotein-like properties in neuroblastoma cells and high HMMR expression has independent prognostic potential in neuroblastomas
Source: Sci Rep. 2025 Nov 18;15:40434. doi: 10.1038/s41598-025-23141-5 (PMC12627537; doi:10.1038/s41598-025-23141-5)
Supplement: Supplementary file 1 — Supplementary Information 1. [file 41598_2025_23141_MOESM1_ESM.docx]

**Legends for Supplementary Files**

**Supplementary Figure S1: (A)** Oncomine platform analysis of the expression of *HMMR* in neuroblastomas compared to ganglioneuroblastomas and ganglioneuromas in Janoueix-Lerosey Brain (top) and Albino Brain (bottom) datasets. The number of tumours analysed for each independent study is also indicated. (**B)** Comparison of the expression of HA axis genes, genes related to hyaluronic acid binding, and cell motility genes in the same neuroblastoma studies as in A examined in Oncomine. Red and blue colours indicate over- and under- expression respectively. Median rank and p-values are also depicted.

**Supplementary Figure S2:** *HMMR* co-expression signature is correlated with poor clinicopathological features in neuroblastoma. (**A)** Overlap of the *HMMR* co-expressed genes in 4 independent neuroblastoma datasets analysed using R2. The 2581 gene list shown in the Venn diagram includes the genes that have significantly similar expression patterns (positive correlation or co-expression) with *HMMR* in four neuroblastoma R2 datasets: Kocak, SEQC, NRC and TARGET-Asgharzadeh. These genes represent the HMMR co-expression signature and were used to generate the heatmap (**B**), the Kaplan-Meier survival analysis using R2 **(C)**, and the GO pathway analysis (**D**). These data were derived from Kocak dataset. The tracks in **B** show age (red > 18 months; green < 18 months); MYCN (red = amplification; green = non-amplification); stage (red = stage 4; blue = stage 4S; brown = stage 3; dark green = stage 2, green = stage 1).Graph in **D** depicts the fold enrichment scores (fdr<0.05).

**Supplementary Figure S3:** DNA sequence traces of KELLY subclones after CRISPR/Cas9 treatment to target *HMMR*. Guide RNA target is underlined. Vertical dashed line is predicted cut site. KC17 has no indels; KA5 has homozygous 1bp inserts; KA14 and KA16 both have the same homozygous 1bp deletions.

**Supplementary Figure S4:** Volcano plots **(A)** and quantification **(B)** of up- and down-regulated phosphosites in KA5 and KA14 cells, respectively, as compared to parental KELLY cells. Red and blue dots correspond to phosphopeptides that changed significantly with respect to control cells (P ≤ 0.05), while black dots represent phosphopeptides outside the filtering criteria (P ≥ 0.05). The number of up- and down-regulated phosphosites in KC17 subclone are also depicted in **B**. (**C)** PCA analysis of the phosphoproteomics data for KELLY, KC17, KA5 and KA14 cells.

**Supplementary Figure S5:** (**A)** Up- and down- regulated kinases of the HMMR phosphoproteomic signature are depicted as a bar plot for both *HMMR* KO lines. Only MAKPK1 and MAPK3 pathways, in both KA5 and KA14, are above the fold-change -Log_10_ P-value threshold of 1.3. (**B)** Visualisation of the MTOR and ERBb components as a protein network. The outer and inner rings represent the log Fc changes in KA5 and KA14, respectively. Red and blue colour indicate increased or decreased log FC, respectively, with greater colour intensity reflecting greater fold changes. (**C)** IPA upstream regulator analysis of the ERK pathway protein components in KA5 (left) and KA14 (right). Data are displayed as a network with ERK illustrated in the center as ‘inhibited’ and surrounded by its downstream targets (nodes). Each molecule is color-coded based on the phosphorylation status in the dataset. Red and green indicate an increase or decrease in phosphorylation respectively. Dotted lines (edges) indicate the indirect relationship between ERK and its targets due to additional intermediate molecules between them (not depicted in the network). Red and blue edges indicate predicted activation and inhibition respectively of the target proteins whereas the yellow and grey lines are depicted when the activity status is contradictory to the prediction or not predicted. Explanation of the color-coding of the predicted relationship is also indicated in the Prediction Legend.

**Supplementary Figure S6:** The ERK1/2 signaling pathway in both *HMMR* KO clones analysed by IPA. The colour coding is the same as described in Supplementary Fig. S5 and indicated in the Prediction Legend.

**Supplementary Figure S7:** Colony formation of HMMR-depleted IMR32 cell subclones.(A) Two examples of 6-well plates stained with crystal violet, 3 weeks after plating of either 500 or 1000 cells. (**B)** Plot of colony counts from n=5 replicates; expressed as a mean ± SD; *p<0.05. (**C)** Immunoblots showing HMMR and GAPDH from the subclones. (**D)** Sequencing traces of the gRNA-targeted region. Note that subclone IB13 is heterogeneous, with 45% +1, 23% -20, 1%. -9, 2% -12, 2% -27 (zero wild type sequence was present), but no detectable protein is observed (panel C).

**Supplementary Figure S8:** Original western gel data for Figure 2B, 6C and Supplementary Fig.S7. The original, whole western filter images are shown with a white box demarcating the dataset used in the respective figures. For each Figure, all of the white-boxed areas are from single sets of lysates, transferred onto a single filter and re-probed with multiple antibodies.
