# Supplementary material for: HMMR has oncoprotein-like properties in neuroblastoma cells and high HMMR expression has independent prognostic potential in neuroblastomas
Source: Sci Rep. 2025 Nov 18;15:40434. doi: 10.1038/s41598-025-23141-5 (PMC12627537; doi:10.1038/s41598-025-23141-5)

Supplementary Figures plus full-sized immunoblots

HMMR has oncoprotein-like properties in cells of neuroblastoma origin and high *HMMR* expression has independent prognostic potential in neuroblastomas.

Christina Karapouliou, Elisyazaviera Muhamad Faizul, Vinothini Rajeeve, Pedro R. Cutillas and Andrew W. Stoker

Figure S1

A

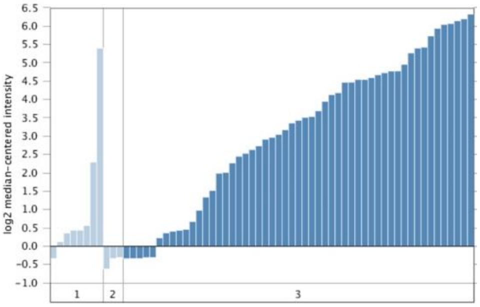

Legend

1. Ganglioneuroblastoma (8)
2. Ganglioneuroma (3)
3. Neuroblastoma (53)

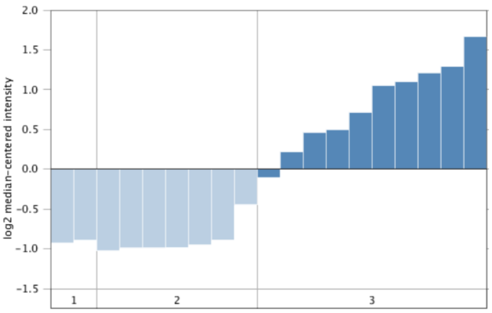

Legend

1. Ganglioneuroblastoma (2)
2. Ganglioneuroma (7)
3. Neuroblastoma (10)

B

Over-expression

Median Rank	p-Value	Gene
413.5	1.92E-4	HMMR
2447.0	0.011	HYAL3
4226.5	0.027	HYAL4
6384.5	0.413	CD44
6940.0	0.392	HAS1
9199.5	0.351	HYAL2
14401.5	0.946	HYAL1
15443.0	0.977	HAS2
-	-	HAS3

1 2

hyaluronic acid binding

Median Rank	p-Value	Gene
413.5	1.92E-4	HMMR
1641.0	0.003	BCAN
3636.0	0.052	ACAN
3762.5	0.019	NCAN
4660.0	0.031	HAPLN1
4964.0	0.036	HAPLN2
6384.5	0.413	CD44
7154.5	0.098	IMPG2
7655.0	0.232	VCAN
14476.0	0.956	LYVE1
15094.5	0.983	TNFAIP6
-	-	HAPLN3
-	-	HAPLN4

1 2

cell motility

Median Rank	p-Value	Gene
413.5	1.92E-4	HMMR
1212.5	0.001	HGFAC
1309.5	0.003	WASF1
1532.0	0.007	TSPAN2
1818.5	0.005	PAK4
1920.5	0.004	AKAP4
1987.5	0.006	KPTN
2017.0	0.004	MAPK8
3268.5	0.022	DNAH9
3328.5	0.027	NTN1
3660.5	0.028	TXN
3762.5	0.019	NCAN
3763.5	0.093	CXCR2
4120.5	0.026	MTSS1
4558.0	0.043	LTB4R
4580.5	0.030	SERPINB5
5031.0	0.037	WASL
5132.5	0.044	MMP12
5246.5	0.088	IL13
5259.5	0.045	MST1R

1 2

1. Neuroblastic Tumor Type: Neuroblastoma
   Albino Brain, Cancer, 2008
2. Neuroblastic Tumor Type: Neuroblastoma
   Janoueix-Lerosey Brain, Nature, 2008

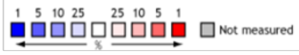

Figure S2

A

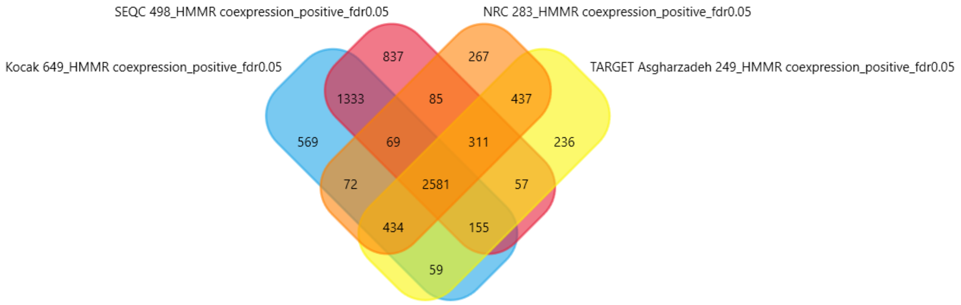

B

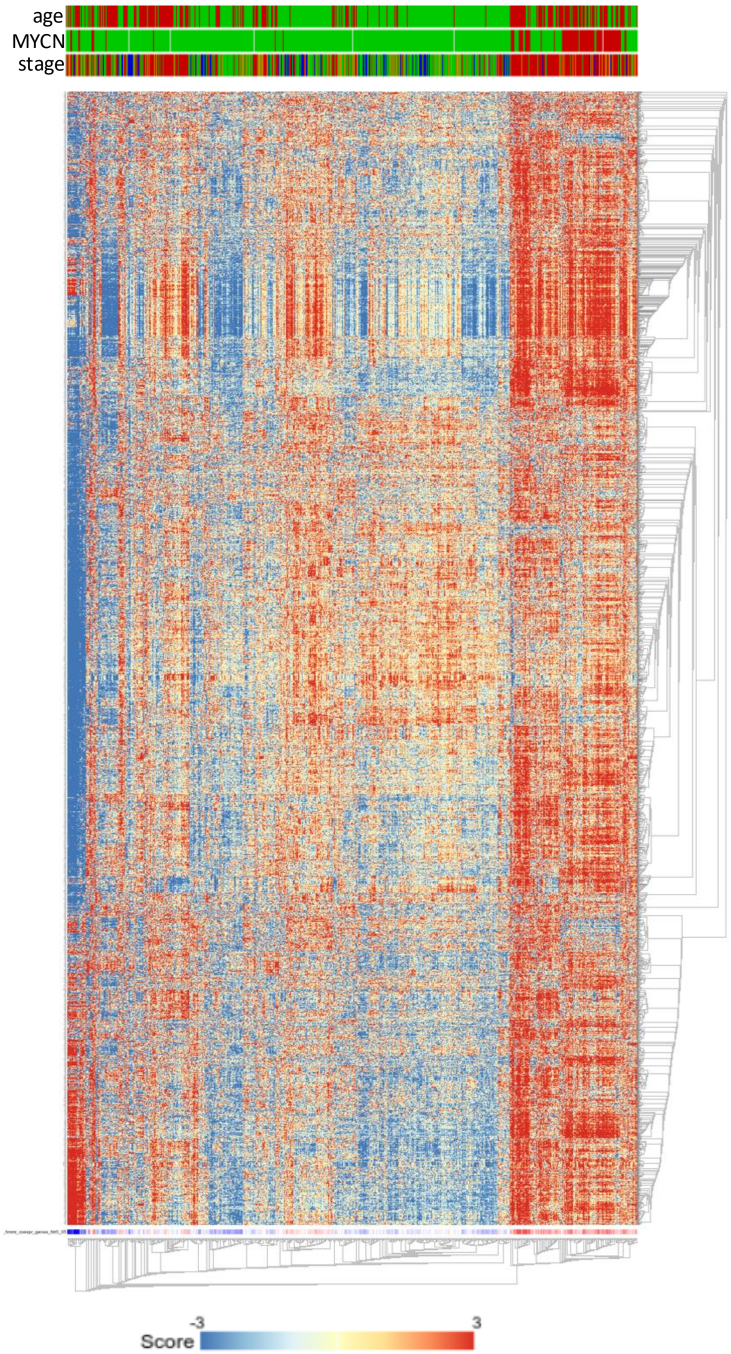

C

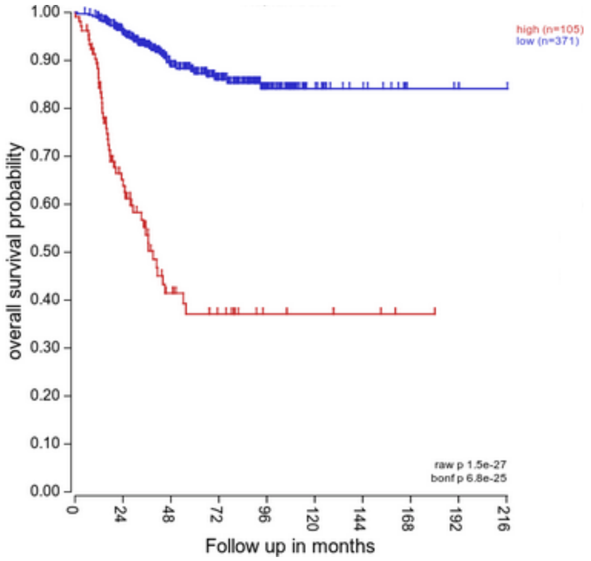

D

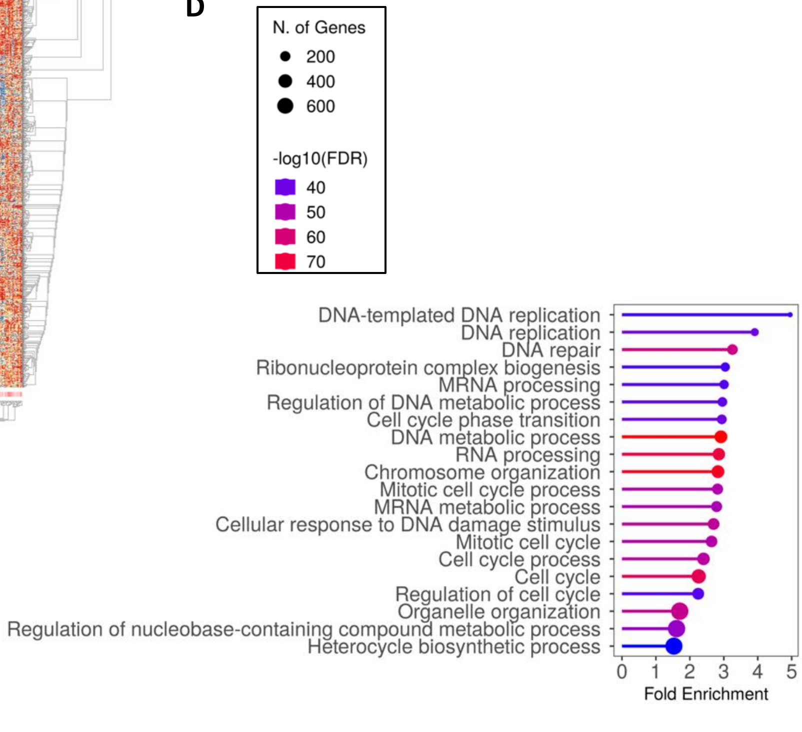

Figure S3

A

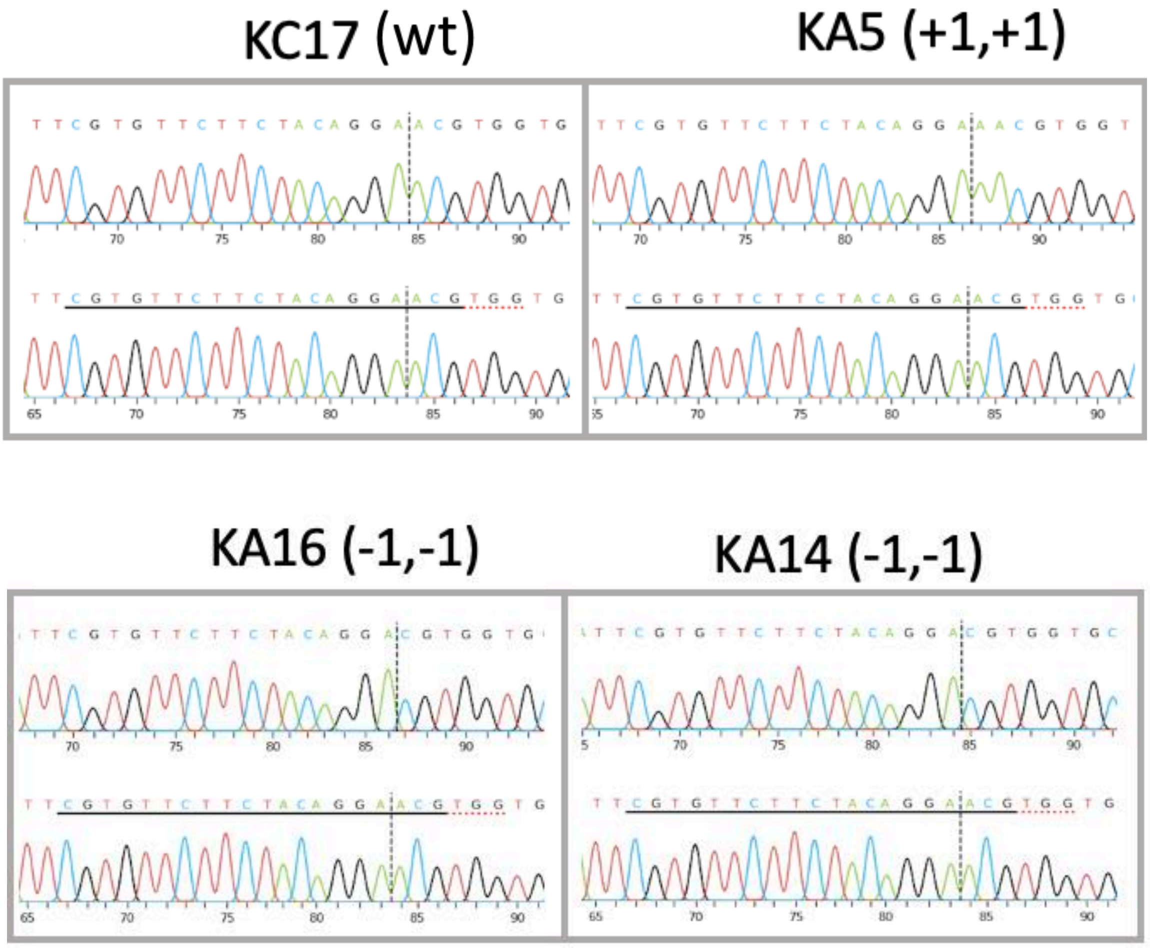

B

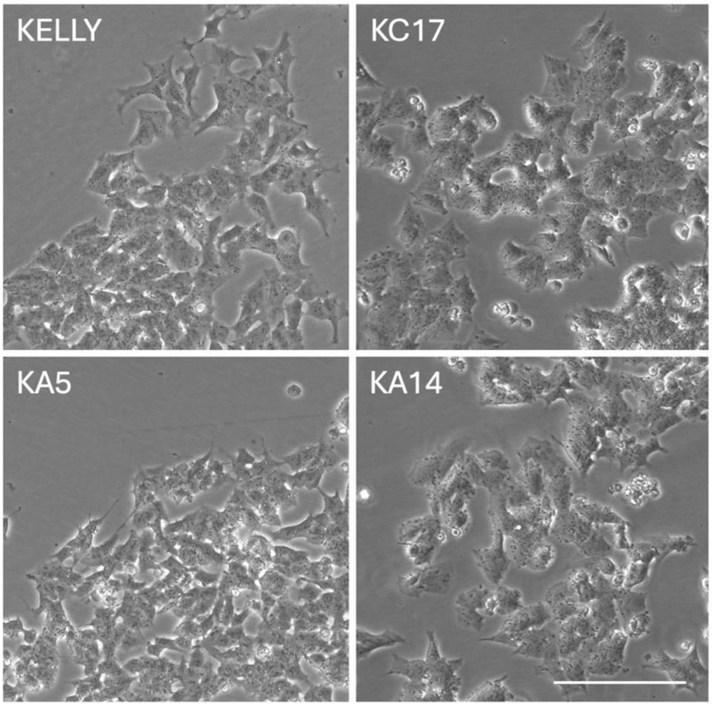

Figure S4

A

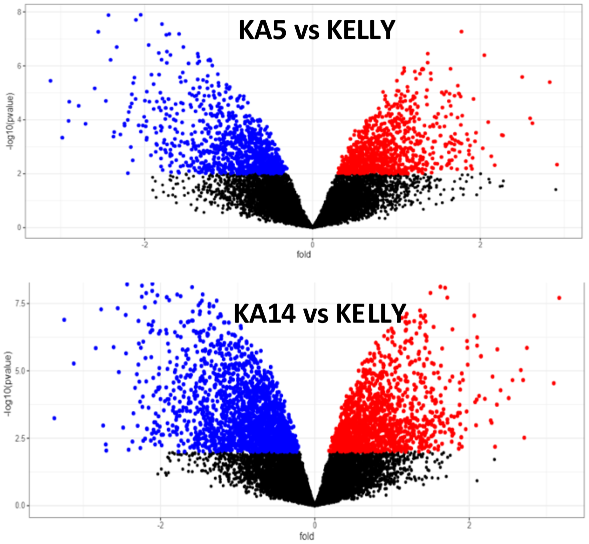

B

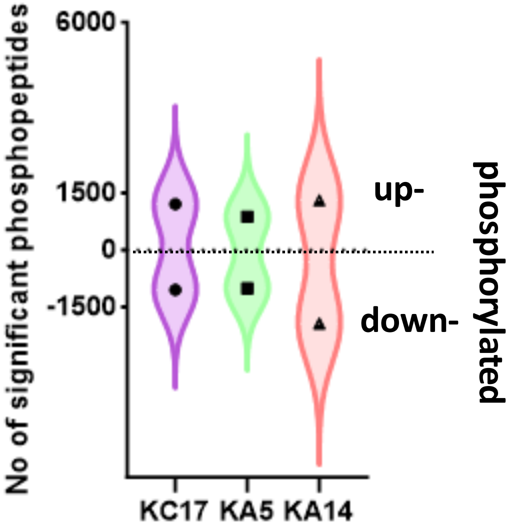

C

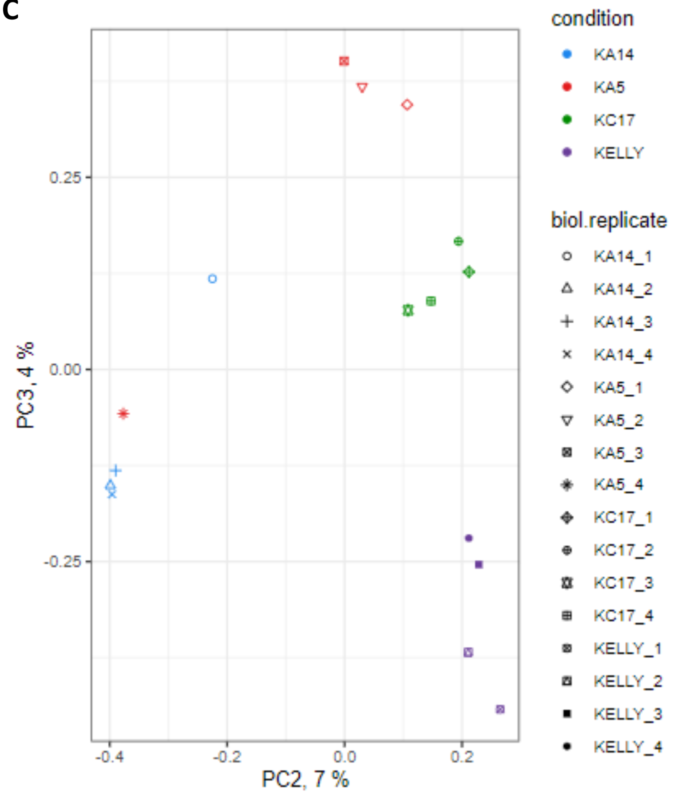

Figure S5

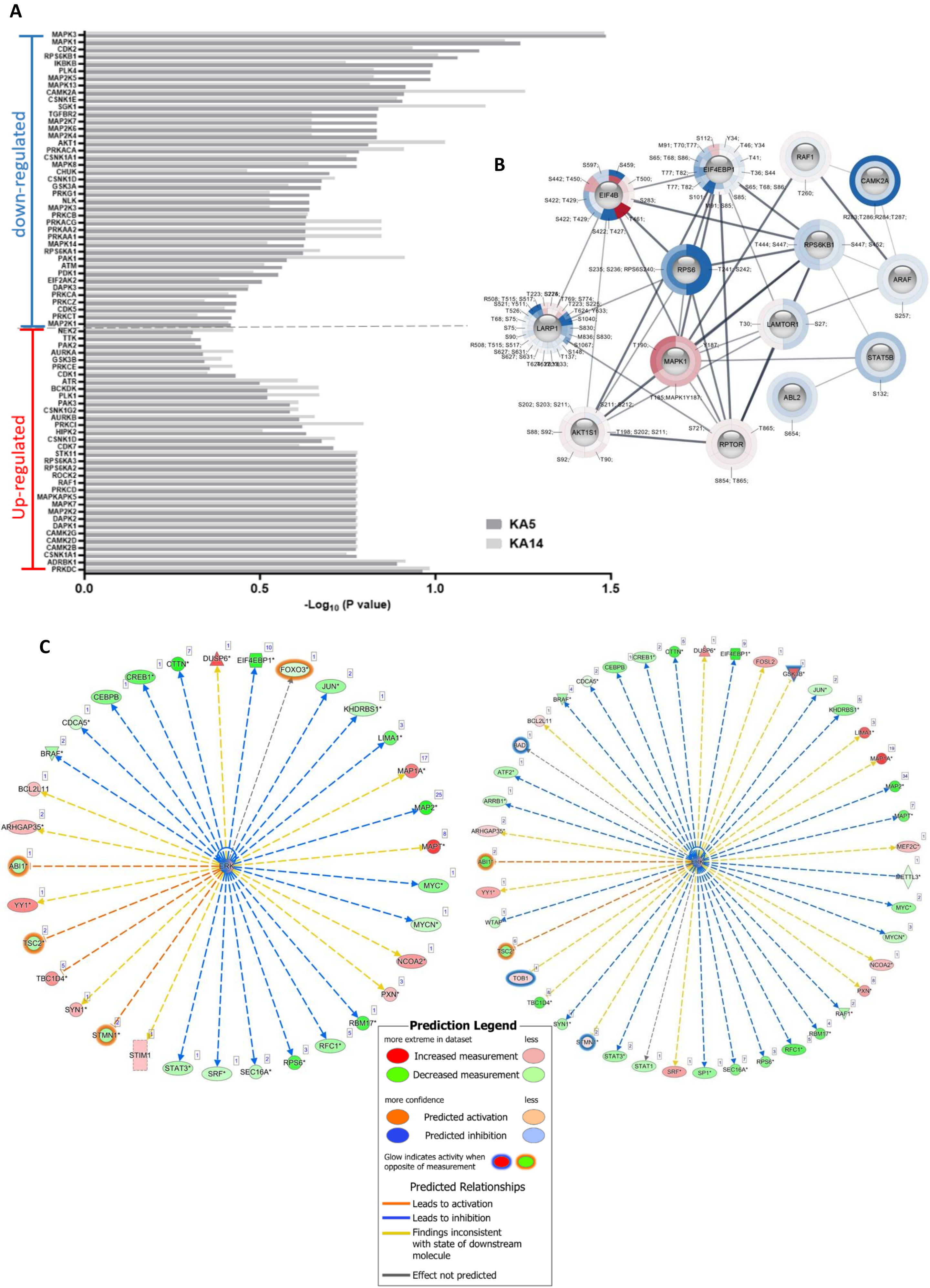

A
down-regulated
Up-regulated
MAPK3
MAPK1
CDK2
RPS6KB1
IKBKB
PLK4
MAP2K5
MAPK13
CAMK2A
CSNK1E
SGK1
TGFBR2
MAP2K7
MAP2K6
MAP2K4
AKT1
PRKACA
CSNK1A1
MAPK8
CHUK
CSNK1D
GSK3A
PRKG1
NLK
MAP2K3
PRKCB
PRKACG
PRKAA2
PRKAA1
MAPK14
RPS6KA1
PAK1
ATM
PDK1
EIF2AK2
DAPK3
PRKCA
PRKCZ
CDK5
PRKCT
MAP2K1
NEK2
TTK
PAK2
AURKA
GSK3B
PRKCE
CDK1
ATR
BCKDK
PLK1
PAK3
CSNK1G2
AURKB
PRKCI
HIPK2
CSNK1D
CDK7
STK11
RPS6KA3
RPS6KA2
ROCK2
RAF1
PRKCD
MAPKAPK5
MAPK7
MAP2K2
DAPK2
DAPK1
CAMK2G
CAMK2D
CAMK2B
CSNK1A1
ADRBK1
PRKDC
0.0
0.5
1.0
1.5
-Log10 (P value)
KA5
KA14
B
EIF4B
EIF4EBP1
RAF1
CAMK2A
RPS6KB1
RPS6
ARAF
LARP1
LAMTOR1
MAPK1
STAT5B
ABL2
AKT1S1
RPTOR
C
ERK
Prediction Legend
more extreme in dataset
less
Increased measurement
Decreased measurement
more confidence
less
Predicted activation
Predicted inhibition
Glow indicates activity when opposite of measurement
Predicted Relationships
Leads to activation
Leads to inhibition
Findings inconsistent with state of downstream molecule
Effect not predicted

Figure S6

KA5

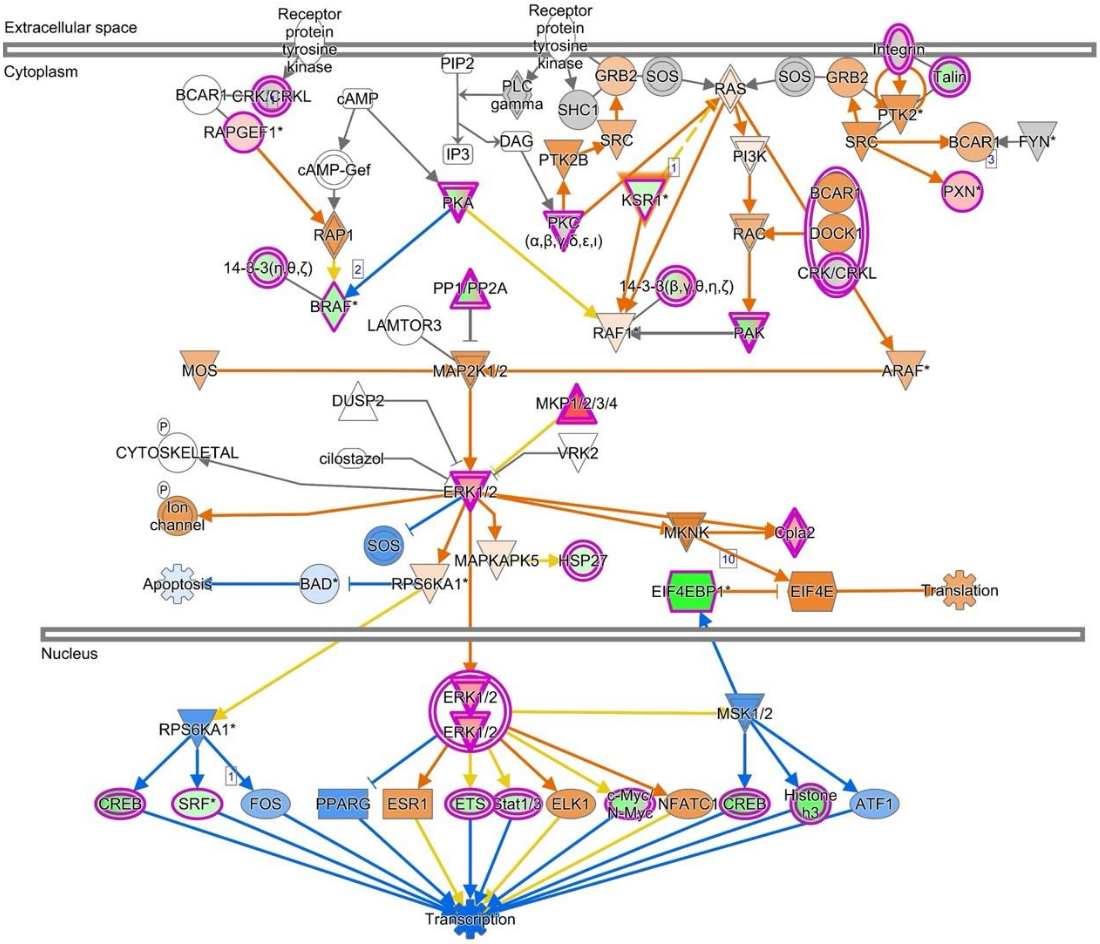

KA14

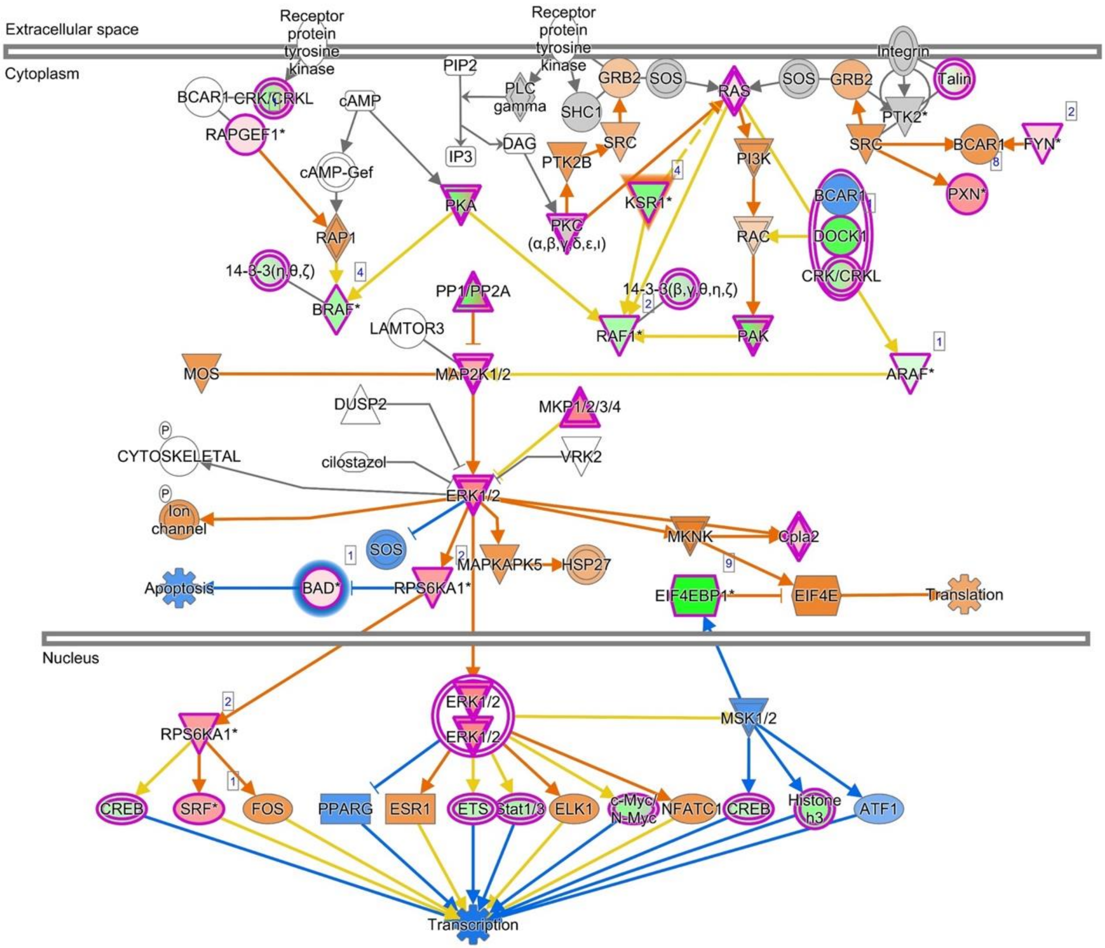

Figure S7

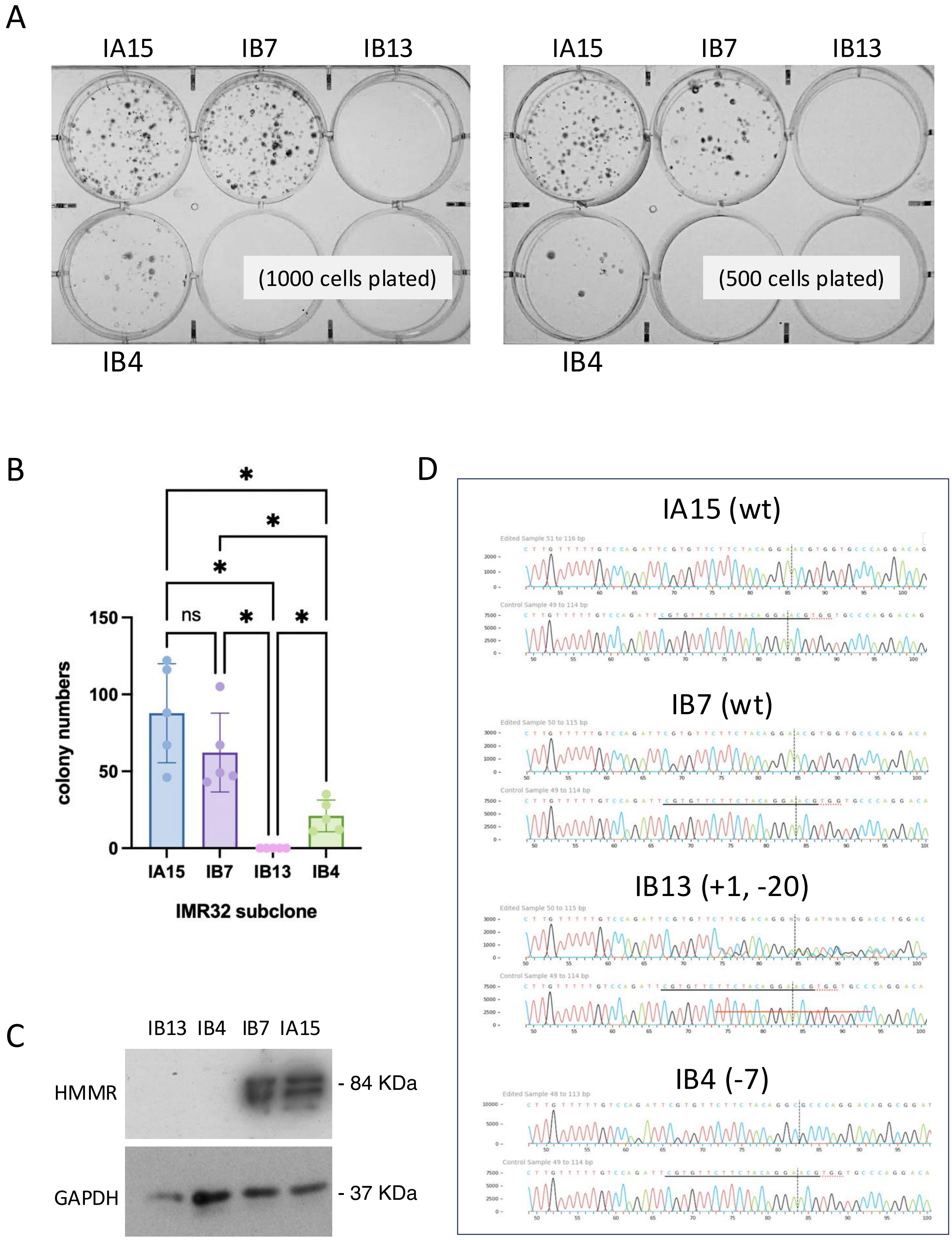

A
IA15
IB7
IB13
IB4
(1000 cells plated)
(500 cells plated)
B
colony numbers
150
100
50
0
ns
*
IA15
IB7
IB13
IB4
IMR32 subclone
C
IB13 IB4 IB7 IA15
HMMR
GAPDH
- 84 KDa
- 37 KDa
D
IA15 (wt)
IB7 (wt)
IB13 (+1, -20)
IB4 (-7)
Edited Sample
Control Sample

Figure S8

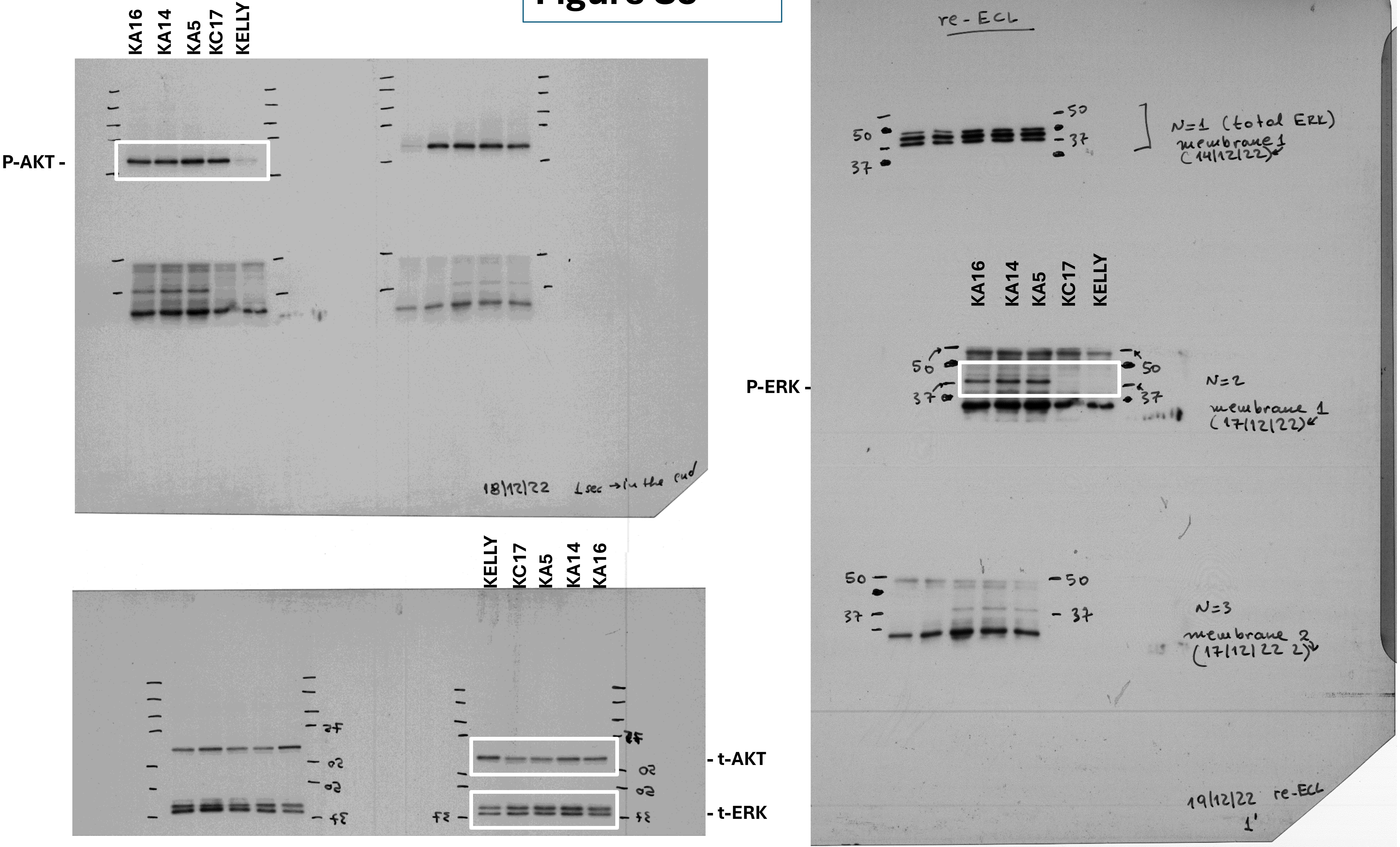

Gels used for panels in Figure 6C

For panels in Figure 6C and Figure 2B

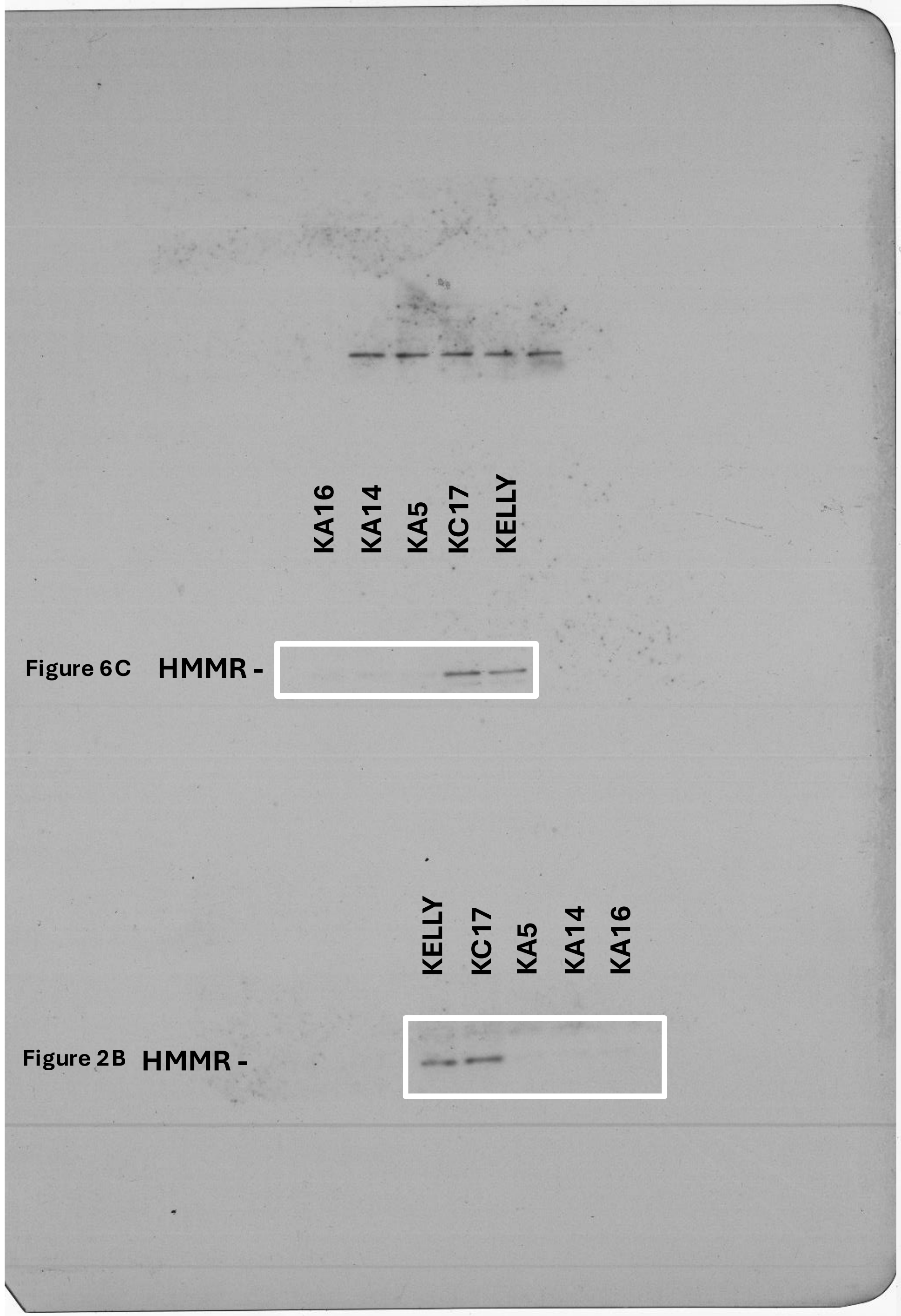

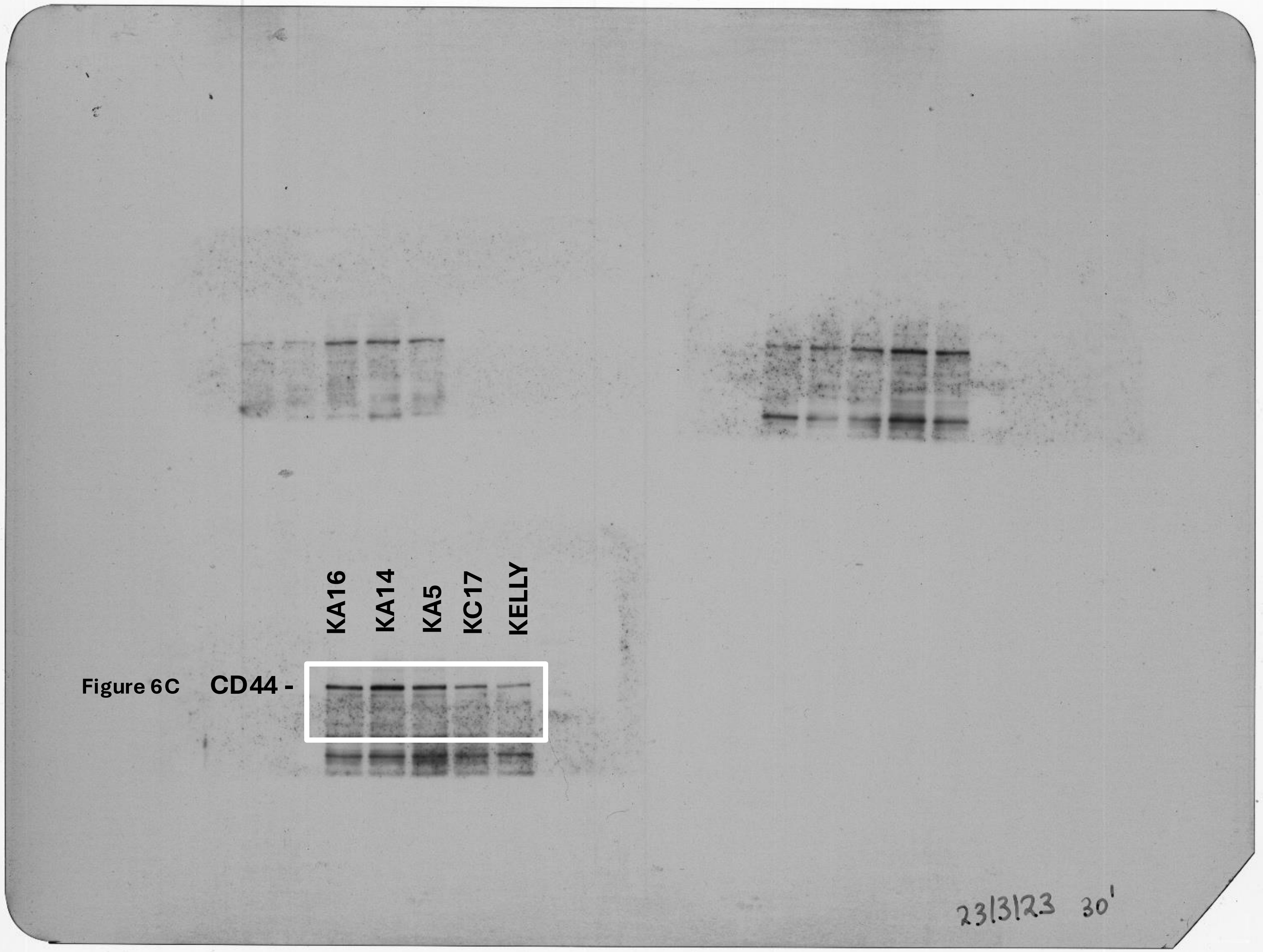

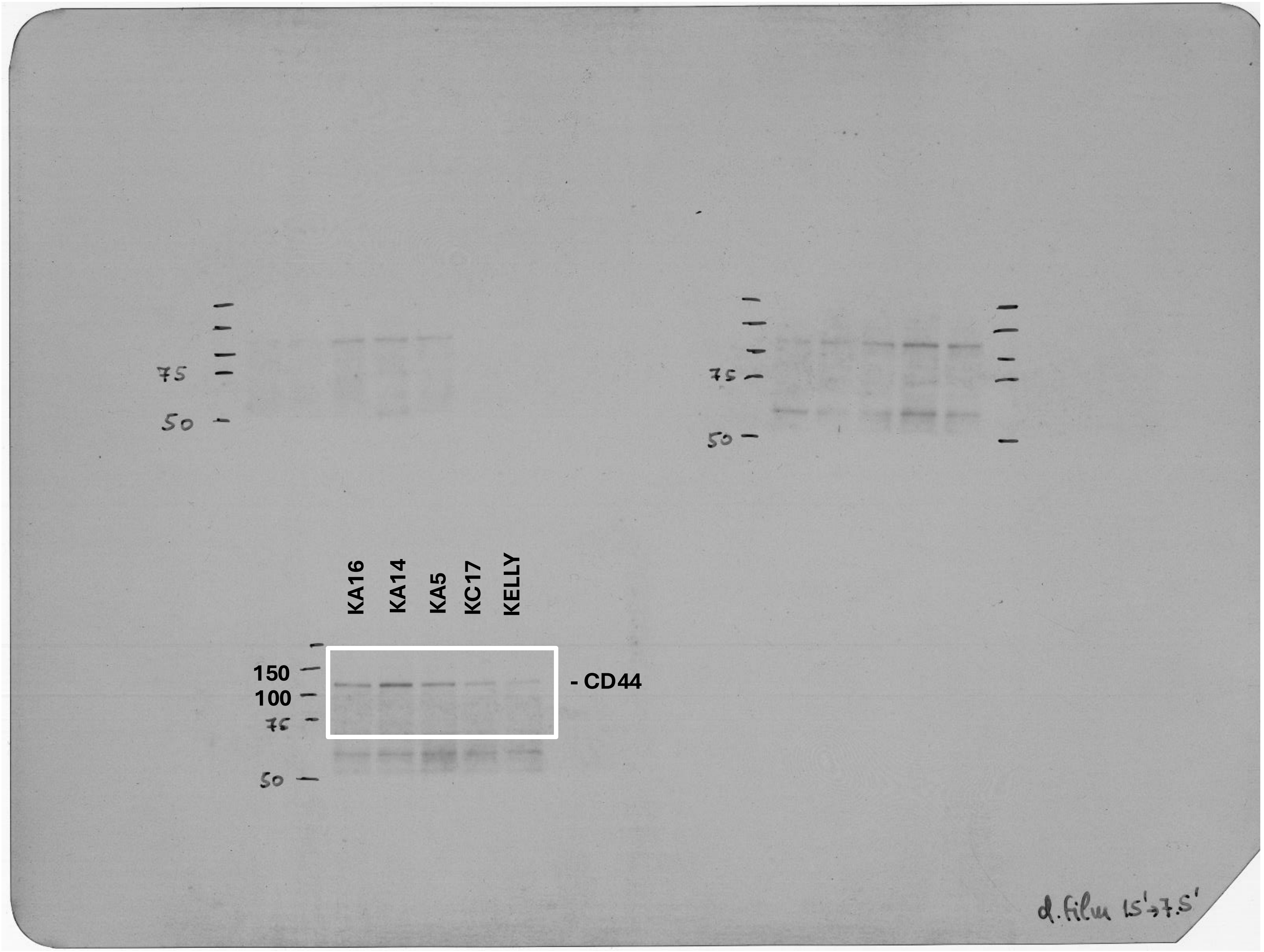

CD44 – uncropped film; lower exposure but with protein marker sizes included – supplementary to Figure 6C

For panels in Figure 6C and Figure 2B

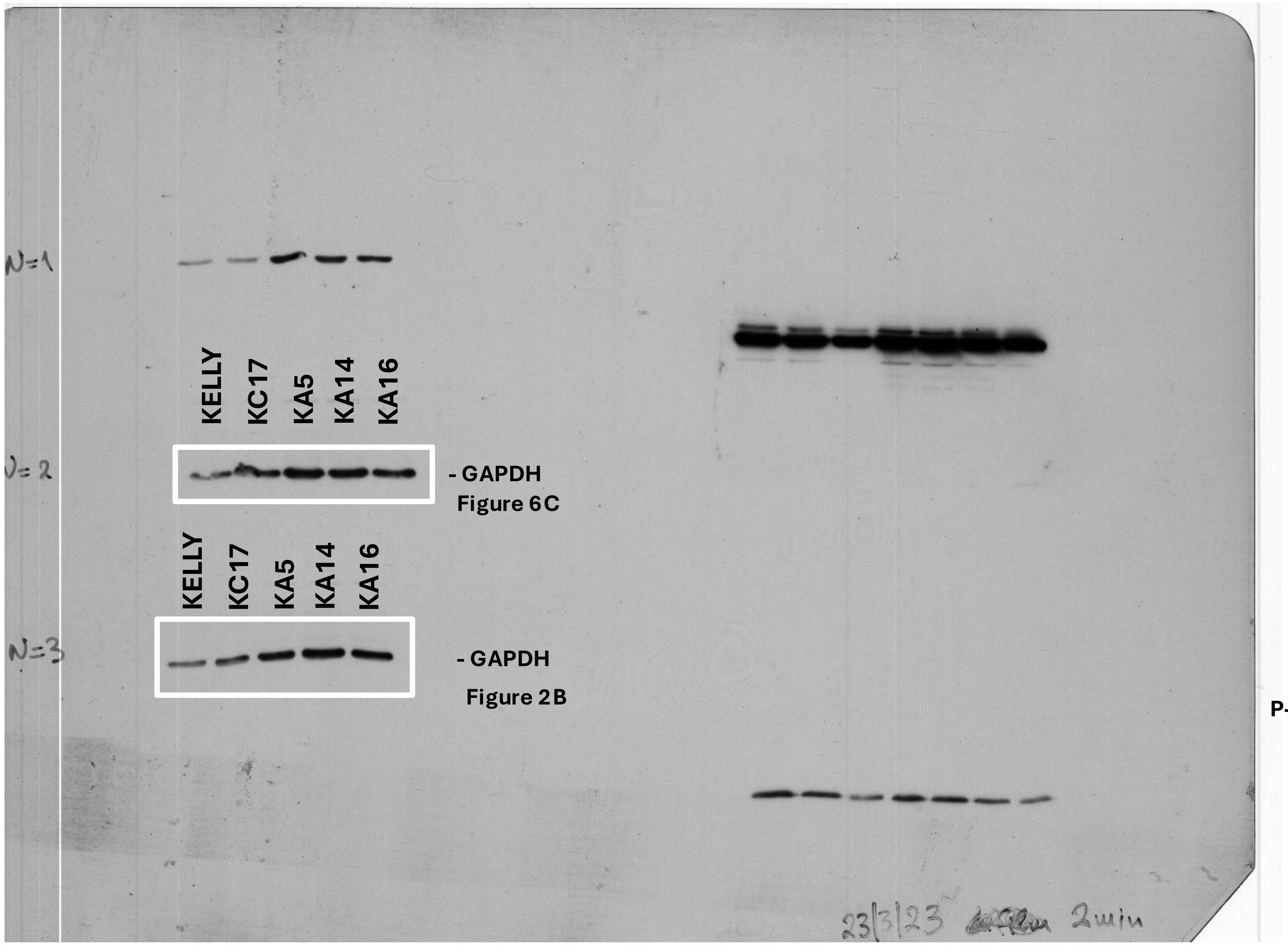

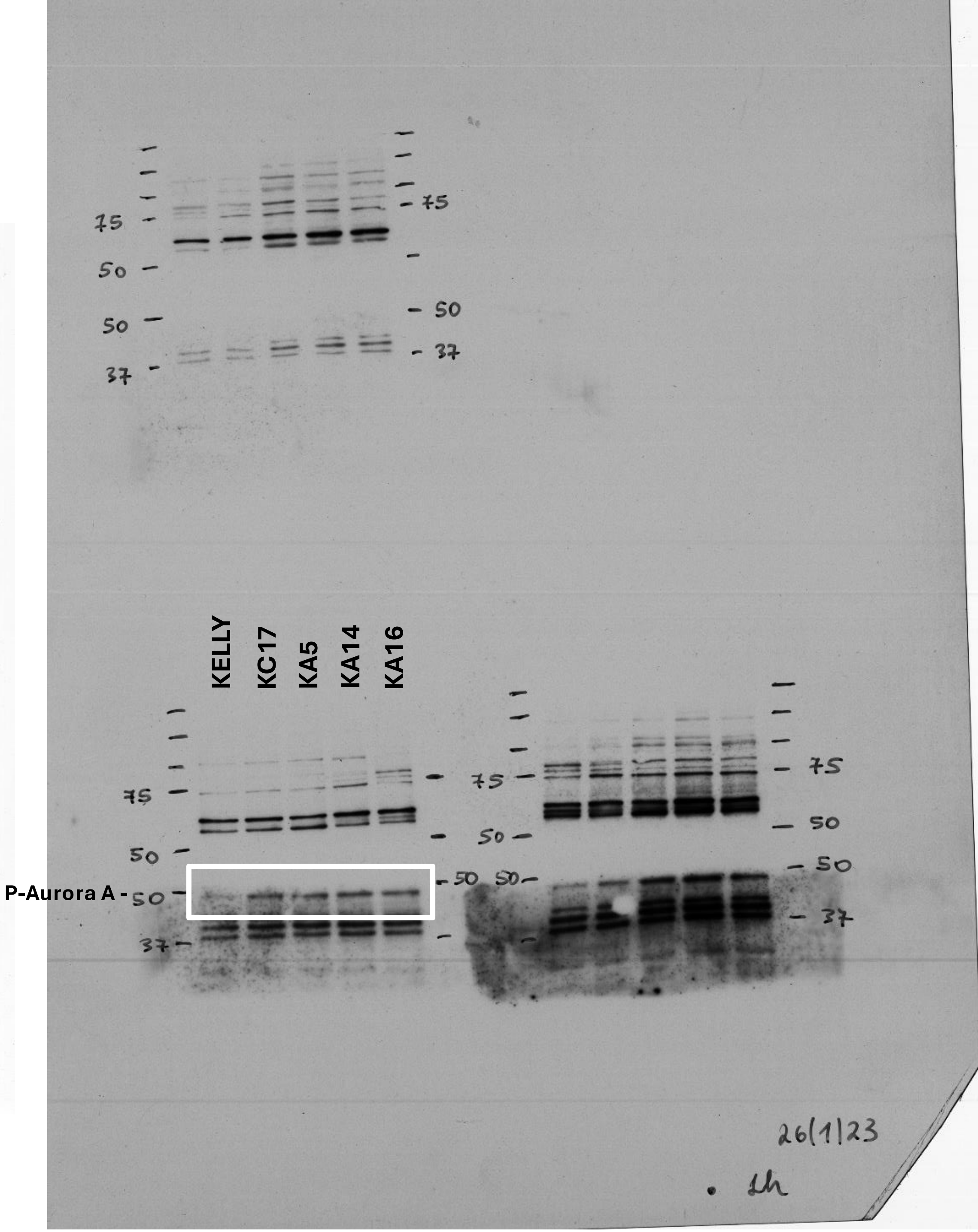

N=2

Construction of Figure 6C

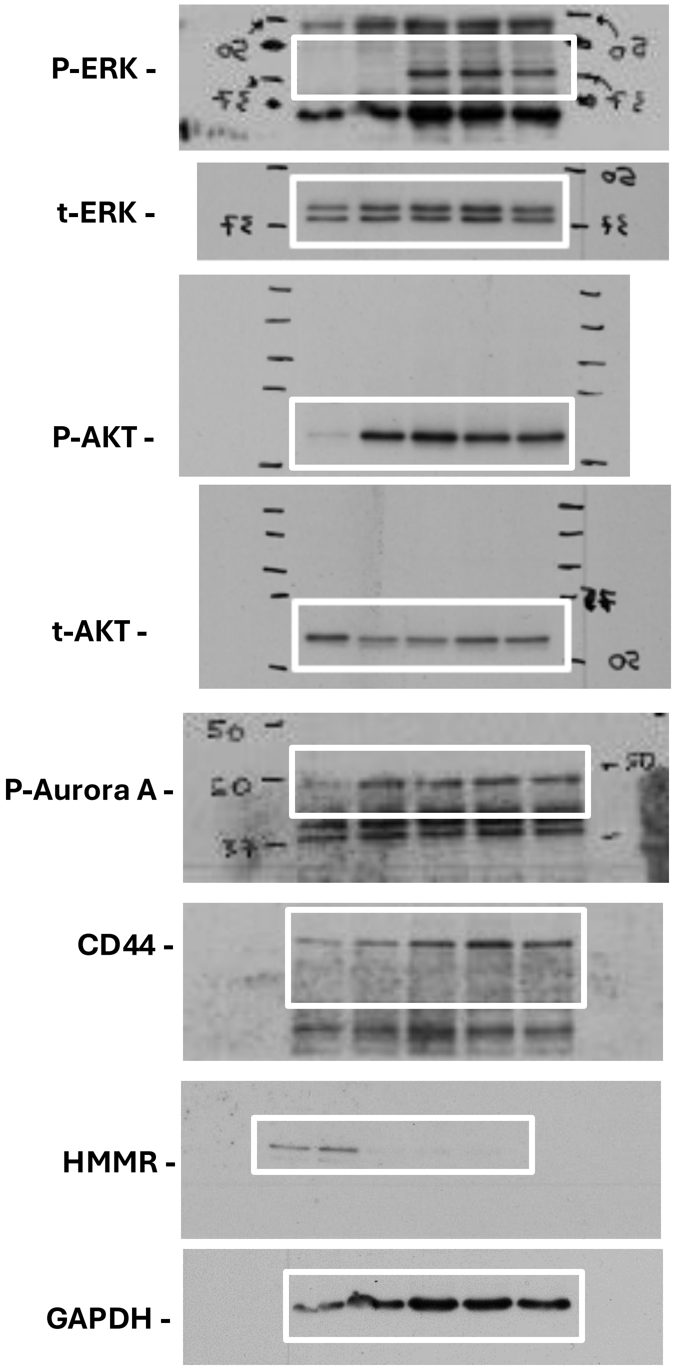

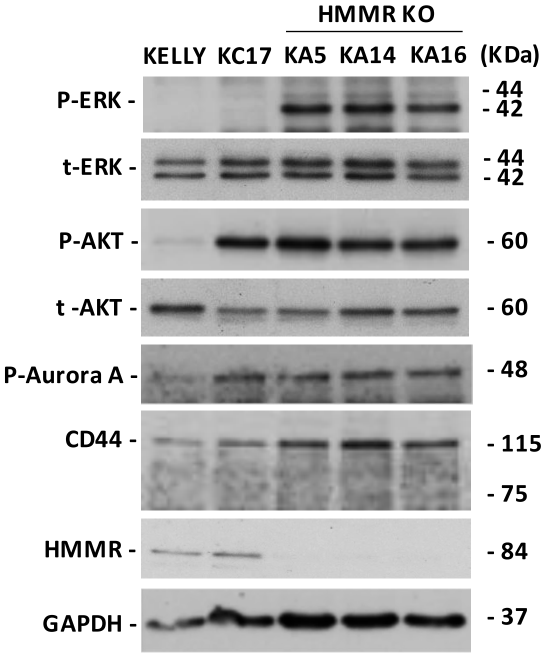

Gels for Figure 2B

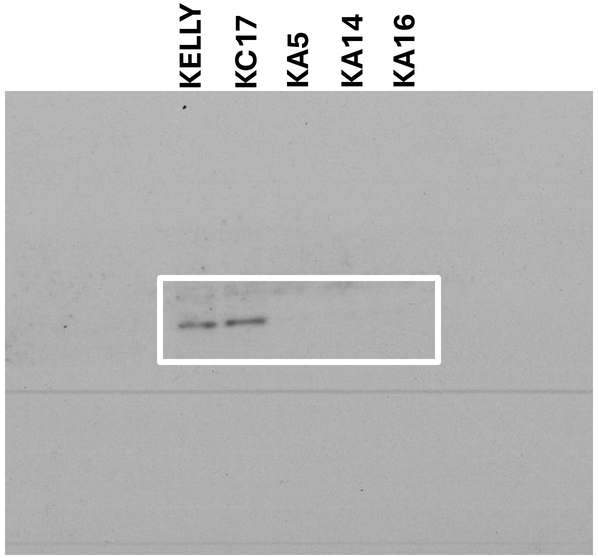

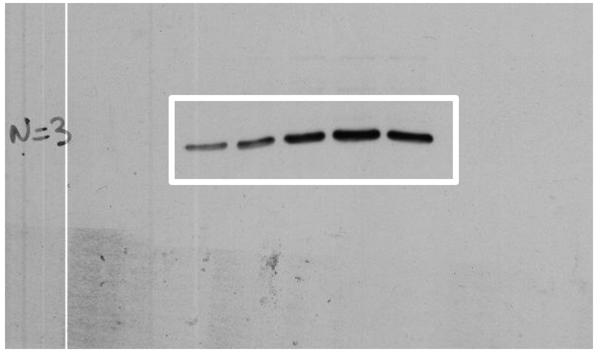

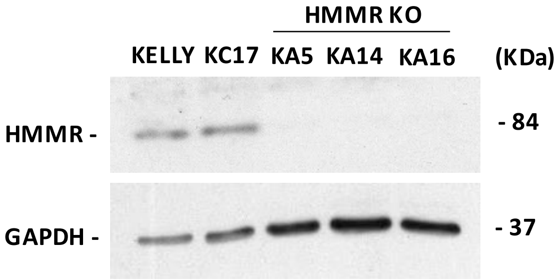

Gels for Supplementary Figure S7

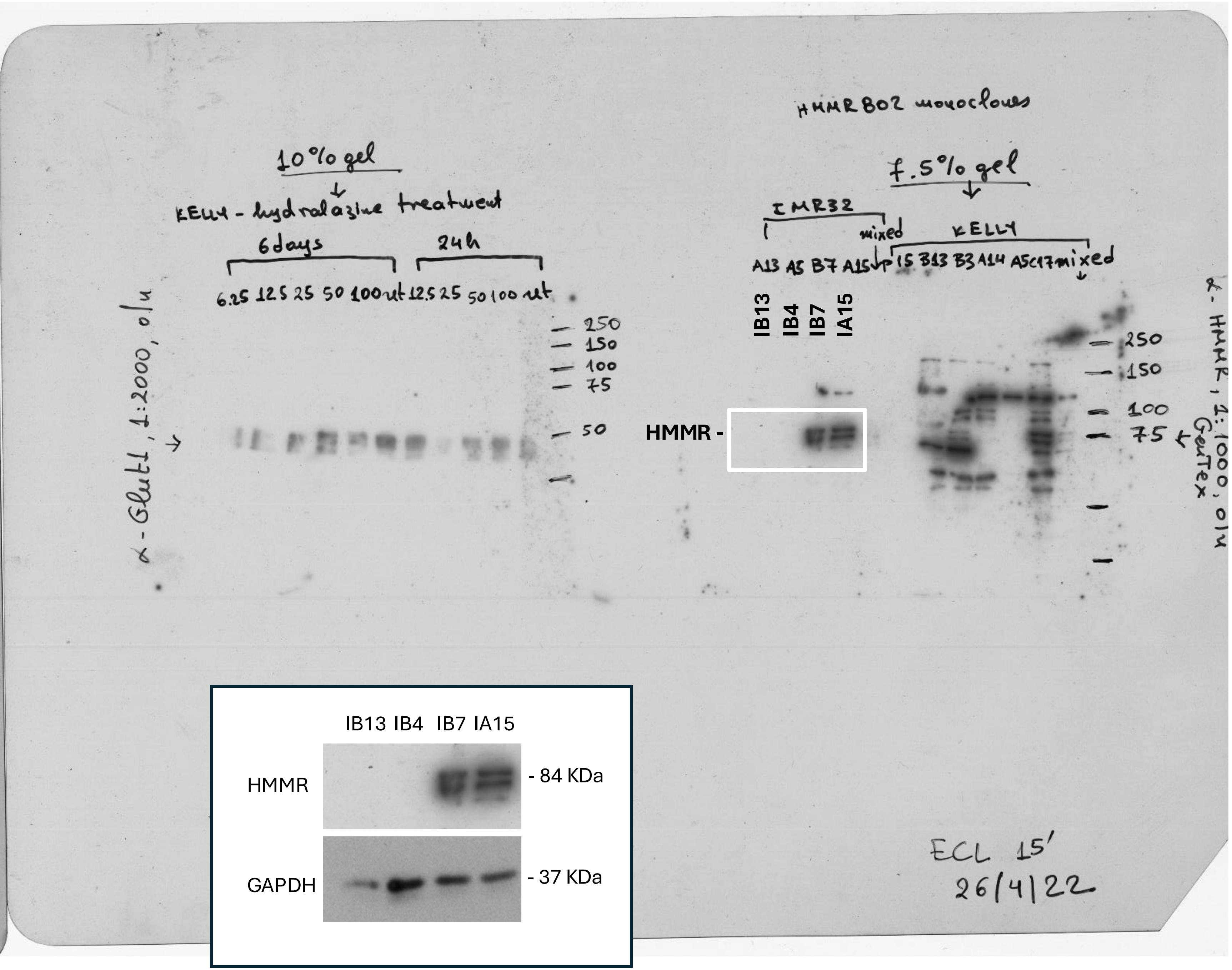

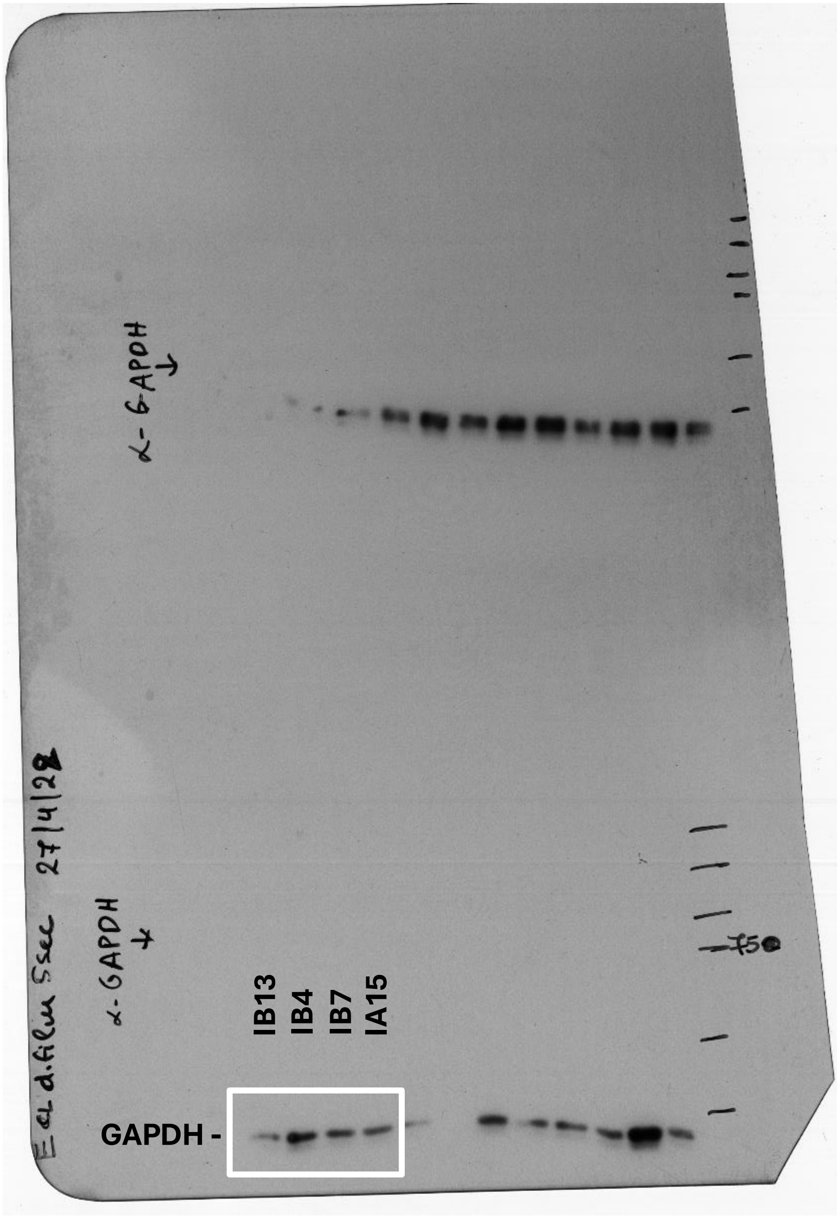

Supplement: Supplementary file 2 — Supplementary Information 2. [file 41598_2025_23141_MOESM2_ESM.pdf]
